# Synthesis, Antiproliferative Activity, and DNA Binding Studies of Nucleoamino Acid-Containing Pt(II) Complexes

**DOI:** 10.3390/ph13100284

**Published:** 2020-09-30

**Authors:** Claudia Riccardi, Domenica Capasso, Angela Coppola, Chiara Platella, Daniela Montesarchio, Sonia Di Gaetano, Giovanni N. Roviello, Domenica Musumeci

**Affiliations:** 1 Department of Chemical Sciences, University of Napoli Federico II, 80126 Napoli, Italy; claudia.riccardi@unina.it (C.R.); angelacoppola94@gmail.com (A.C.); chiara.platella@unina.it (C.P.); montesar@unina.it (D.M.); 2 CESTEV, University of Napoli Federico II, 80145 Napoli, Italy; domenica.capasso@unina.it; 3 CNR, Institute of Biostructure and Bioimaging, 80134 Napoli, Italy; digaetan@unina.it (S.D.G.); giroviel@unina.it (G.N.R.)

**Keywords:** Pt(II) complexes, nucleoamino acid ligand, NMR spectroscopy, antiproliferative activity, DNA binding studies, CD spectroscopy

## Abstract

We here report our studies on the reaction with the platinum(II) ion of a nucleoamino acid constituted by the l-2,3-diaminopropanoic acid linked to the thymine nucleobase through a methylenecarbonyl linker. The obtained new platinum complexes, characterized by spectroscopic and mass spectrometric techniques, were envisaged to exploit synergistic effects due to the presence of both the platinum center and the nucleoamino acid moiety. The latter can be potentially useful to protect the complexes from early deactivation, as well as to facilitate their cell internalization. The biological activity of the complexes in terms of antiproliferative effects was evaluated in vitro on different cancer cell lines and healthy cells, showing the best results on human cervical adenocarcinoma (HeLa) cells along with good selectivity for cancer over normal cells. In contrast, the metal-free nucleoamino acid did not show any cytotoxicity on both normal and cancer cell lines. Finally, the ability of the novel Pt(II) complexes to bind various DNA model systems was investigated by circular dichroism (CD) spectroscopy and polyacrylamide gel electrophoresis analyses proving that the newly obtained compounds can potentially target DNA, similarly to other well-known anticancer Pt complexes, with a peculiar G-quadruplex vs. duplex selectivity.

## 1. Introduction

Cisplatin (cis-diaminedichloro-platinum, CDDP) is one of the most active chemotherapeutic agents used for the treatment of a large variety of malignancies, especially testicular and ovarian carcinomas, even if its clinical utility is restricted by both toxicological and tumor resistance drawbacks [1,2]. CDDP activity and toxicity is mainly due to DNA binding [3,4], occurring via both inter- and intrastrand crosslinks, that suppresses DNA replication and also RNA transcription. Specifically, CDDP interacts with the DNA nucleobases forming stable platinum–nitrogen covalent bonds, which lead to irreversible adducts responsible for cisplatin antitumor activity [1,4].

The drawbacks and side effects of cisplatin treatment [1,2] have stimulated the design and synthesis of new anticancer Pt-based drugs with the aim of obtaining compounds more effective and less toxic than CDDP. Thus, in the last decades, a wide variety of Pt complexes have been tested for antiproliferative activity [5,6,7]. Some have also entered in clinical trials, but only a few have been approved for clinical use.

**Figure 1:**
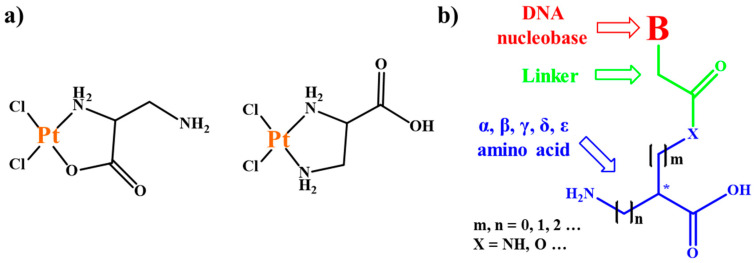
(**a**) Platinum complexes of l-2,3-diaminopropanoic acid (DAP-OH); (**b**) general structure of a nucleoamino acid.

As ligands to be incorporated in Pt-based complexes, most studies focused on bioactive molecules, such as peptides [8], carbohydrates [7,9], fatty acids [10,11], amino acids (both proteinogenic or not) [8,12], nucleosides, nucleotides [13,14,15], and natural products [16], to obtain compounds with enhanced activity thanks to the synergistic effects of the Pt ion and the bioactive ligand. Since the aforementioned bioactive molecules are widespread in living organisms, when used as Pt(II) ligands, the metal center can be masked, retarding the complex deactivation or avoiding its recognition by a specific protein. Nucleosides or nucleobases represent an interesting class of molecules for drug conjugation, due to the presence in vivo of specific proteins which can favor their delivery and cellular internalization [17,18]. Thus, their conjugation to a reactive Pt center can produce molecules with remarkable biological properties. On the other side, amino acids may bind Pt(II) ions as bidentate chelates, providing a five-membered ring, including the platinum center, the α-amino, and the α-carboxylic acid functions [8,19,20]. Furthermore, polar and charged amino acids may also coordinate platinum through their side chains [19]. The nature and length of the side chains can also influence the solubility and membrane permeability of the resulting complexes and, consequently, the platinum concentration inside the cell [21]. It is interesting to consider the reaction between the metallic center and diamino acids, or, in general, amino acids containing in their side chains amino (lysine, arginine, and histidine) or amide (asparagine and glutamine) groups, that also exploit the side-chain function for the metal chelation [19,22,23]. Complexes with non-proteinogenic amino acids have also been realized, as in the case of the diamino acids ornithine, which binds platinum through the α-NH_2_ and α-COOH functions [8,22], and l-2,3-diaminopropanoic acid (DAP-OH), yielding Pt(II) complexes with a square planar structure in which both the α- and β-amino groups of the diamino acid, along with the α-amino and α-carboxylic functions, can bind the Pt ion forming a 5-membered ring (Figure 1a) [21,22,24].

Considering the capability of the platinum ion to bind both nucleobases and amino acids, our novel design consisted of Pt(II) complexes, incorporating as ligands nucleoamino acids, constituted by nucleobases linked to an amino acid through a suitable linker (Figure 1b), and thus, intrinsically containing various potential platinum binding sites. To the best of our knowledge, this is the first report on Pt complexes incorporating this kind of ligand. Nucleoamino acids have been widely explored in the literature, mainly as building blocks for the assembly of nucleopeptides, compounds potentially able to bind complementary nucleic acids in antisense/antigene strategies, or to give ordered self-assembly, a useful property in the context of the development of new nanomaterials [25,26,27,28,29,30,31].

Recently, a 9-fluorenylmethoxycarbonyl (Fmoc)-protected nucleoamino acid, obtained connecting the Fmoc-diaminopropanoic acid (Fmoc-DAP-OH) to the thymine acetic acid, was synthesized in our group and then used to obtain, via solid-phase peptide synthesis, a homothymine nucleopeptide for potential biomedical applications [30].

Here we used the deprotected DAP(T)–OH nucleoamino acid for platinum complexation to obtain new Pt complexes in which synergistic anticancer effects were envisaged for the presence of both the nucleobase and the platinum center. The selected nucleoamino acid contained several donor groups for platinum (i.e., the nitrogen atoms of the α-amino group, the nucleobase, or the amide in the methylenecarbonyl linker, and the oxygens of the carboxylic function of the nucleoamino acid) and could potentially form a large variety of Pt complexes. The compounds obtained from the platination reaction of DAP(T)–OH were purified and characterized by nuclear magnetic resonance (NMR), mass spectrometry, and ultraviolet–visible (UV-vis) techniques. Then, the antiproliferative activity of the new Pt(II) complexes was evaluated in preliminary tests on different cancer cell lines and normal human cells, using the starting nucleoamino acid as a reference compound. Finally, in analogy to other metal complexes, the ability of the new Pt(II) complexes to bind DNA was also explored using circular dichroism (CD) and polyacrylamide gel electrophoresis analyses on different DNA model systems, both in random coil and in structured forms.

## 2. Results and Discussion

### 2.1. Preparation of the Nucleoamino Acid Ligand

**Scheme 1:**
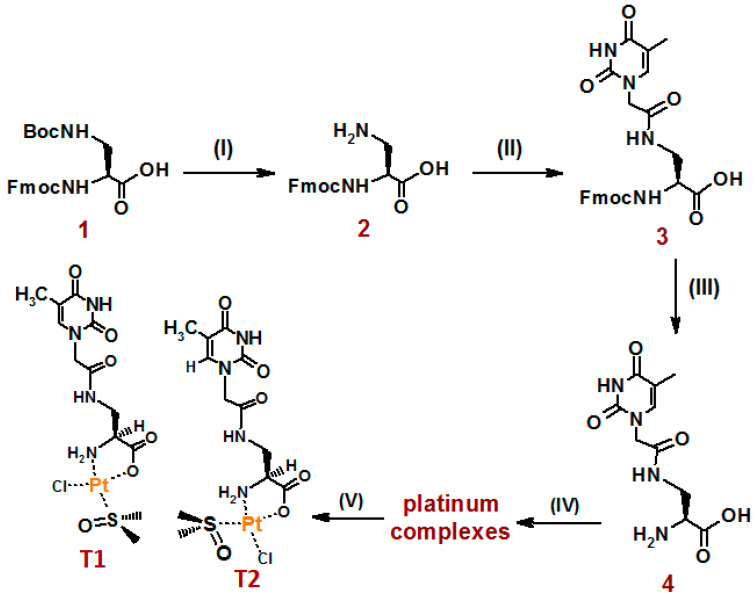
Synthetic procedure for the preparation of the nucleoamino acid DAP(T)–OH(**4**) and its platinum complexes. Reagents and conditions: (I) trifluoroacetic acid (TFA)/CH_2_Cl_2_ (1:1, *v*/*v*), 45 °C, 1.5 h (98% yield); (II) thymine acetic acid, 1-[bis(dimethylamino)methylene]-1H-1,2,3-triazolo[4,5-b]pyridinium 3-oxid hexafluorophosphate/N,N-diisopropylethylamine (HATU/DIEA) in N,N-dimethylformamide (DMF), 2 h, room temperature (r.t.) (80%); (III) piperidine (8 equiv.) in DMF/CH_3_OH (9:1, *v*/*v*), r.t., 10 min (98%); (IV) K_2_PtCl_4_ in CH_3_OH/H_2_O (9:1, *v*/*v*), 6 h, r.t., in the dark; (V) dimethyl sulfoxide (DMSO) dissolution of the platinum complexes obtained under conditions IV to favour DMSO-exchange reaction.

The Fmoc-protected nucleoamino acid **3,** based on l-2,3-diaminopropanoic acid and carrying the nucleobase thymine attached through a methylenecarbonyl linker to its β-amino group, was obtained as previously reported [30]. In brief, commercially available Fmoc-L-DAP(Boc)-OH (**1**, Scheme 1) was first treated with a trifluoroacetic acid (TFA)/CH_2_Cl_2_ (1:1, *v*/*v*) solution for the selective removal of the tert-butoxycarbonyl (Boc) protecting group from the β-amino function (almost quantitative yield). Then, the desired product **2** was coupled with thymine acetic acid using 1-[bis(dimethylamino)methylene]-1H-1,2,3-triazolo[4,5-b]pyridinium 3-oxid hexafluorophosphate/N,N-diisopropylethylamine (HATU/DIEA) as the coupling agent, obtaining compound **3** in 80% overall yield. Fmoc removal from the DAP-moiety in **3** was quantitatively achieved upon treatment with piperidine. The final deprotected nucleoamino acid **4** was purified by high-performance liquid-chromatography (HPLC) and characterized by mass spectrometry (MS), i.e., matrix-assisted laser desorption ionization-time of flight (MALDI-TOF) and liquid chromatography-electrospray ionization (LC-ESI) MS (Figure S1), and NMR spectroscopy (Figures S2 and S3), which in all cases confirmed the identity and purity of the target compound.

### 2.2. Platination of the DAP(T)-Based Nucleoamino Acid

Analogously to previous works [5,12], the platination reaction was carried out mixing DAP(T)–OH (**4**, Scheme 1), as a Pt chelator, and K_2_PtCl_4_ in CH_3_OH:H_2_O (9:1, *v*/*v*) under stirring at room temperature in the dark. Subsequently, the solvent was removed under vacuum at low temperature, and the dried crude was sequentially treated with various organic solvents (see Materials and Methods) to dissolve the yellow solid. In detail, a solid–liquid extraction, followed by washings and centrifugations, using not significantly coordinating solvents with increasing polarity (CH_2_Cl_2_, CHCl_3_, CH_3_COCH_3_, CH_3_OH, H_2_O), was performed. Neither the apolar solvents (CH_2_Cl_2_, CHCl_3_) nor acetone were able to dissolve the solid allowing the recovery of the Pt complexes. In contrast, methanol was able to solubilize and recover a yellow product and, subsequently, H_2_O dissolved the second and last complex (see Materials and Methods).

The two obtained products were subjected to LC-ESI-MS analysis, showing a single chromatographic peak in the total ion current (TIC) profile of the injection performed immediately after their dissolution (t = 0, Figure S4a, left) with a complex mass spectrum denoting fragmentation during the ionization process (Figure S4a, right). Both the complexes showed almost the same peaks in the mass spectrum, evidencing that they followed the same fragmentation pattern and could probably be isomers (as an example, in Figure S4, we reported the LC-ESI-MS analysis of the methanol-recovered complex). From the analysis of the mass peaks of the complexes (Figure S4a, right), we could hypothesize that they both contained in their structures a DAP(T)–OH unit, platinum(II) and chloride ions, and a water molecule. The injection performed 10 min after the dissolution of both the compounds showed at least three peaks in the TIC profiles, each associated with a mass spectrum very difficult to interpret, evidencing that the Pt complexes are very unstable in aqueous solutions (Figure S4b) and, thus, difficult to handle for both their characterization and subsequent in vitro assays.

Then, since the most commonly used NMR organic solvents (CDCl_3_, CD_3_OD, CD_3_COCD_3_) were not able to solubilize the two products at the minimum concentration required for a complete NMR characterization, we decided to dissolve the complexes in a highly solubilizing solvent, such as N,N-dimethylformamide (DMF) or dimethyl sulfoxide (DMSO). Even if these solvents are well known to trigger exchange reactions with various Pt(II) ligands (see [5,15] for DMSO and [13] for DMF), in many cases, DMSO moieties were intentionally inserted in various bioactive platinum complexes to increase their activity and stability [5,15]. The compounds obtained after DMSO treatment (Scheme 1) were named **T1** and **T2**. They were derived, respectively, from the Pt complexes originally recovered with CH_3_OH and H_2_O.

As expected, in the ESI mass spectra (positive ion-mode) of the DMSO-solubilized complexes, we effectively verified the occurrence of the exchange reaction realized by the solvent. Indeed, for both complexes, the found mass peaks were attributed to molecules containing a DAP(T)–OH unit, a platinum(II) ion, a chloride ion, and a DMSO molecule (*m*/*z* ca. 579, Figure S5a), indicating that the two complexes were either constitutional or geometrical isomers. In the recorded mass spectra, **T1**- and **T2**-adducts with Na^+^ and K^+^ ions were also found, respectively, at ca. 601 and 617 *m*/*z* (Figure S5a).

The peak isotopic distribution for each species found in the mass spectra of the two isomers was almost superimposable to the theoretical one calculated with the Isotope Distribution Calculator program (http://www.sisweb.com/mstools/isotope.htm). As an example, we reported the comparison between the theoretical and experimental peak at ca. 579 *m*/*z* of **T1** (Figure S5b).

To verify how the nucleoamino acid ligand coordinated the platinum(II) ion in these isomers, mono- and bi-dimensional NMR experiments were carried out, recording all the spectra in DMSO-d_6_ (Figures S6–S8). The ^1^H and ^13^C-NMR spectra of DAP(T)–OH were used as references to evaluate the shifts of the corresponding signals in the complexes, thus obtaining information on the atoms directly involved in the coordination or influenced in their spectral properties by the proximity to the metal center. In Table 1, the ^1^H and ^13^C NMR chemical shift values for **T1** and **T2** were reported in comparison to the starting ligand. Assignments were based on correlation spectroscopy (COSY), heteronuclear single quantum correlation (HSQC), and heteronuclear multiple-bond correlation (HMBC) experiments (Tables S1–S3).

In particular, in the ^1^H NMR spectra of **T1** and **T2** complexes, two signals, i.e., broad multiplets at 5.68 and 6.29 ppm (for **T1**, Figure S6, up) and 5.63 and 6.25 ppm (for **T2**, Figure S7), were diagnostic of protons belonging to a nitrogen bound to platinum(II) center, in accordance with other complexes reported in the literature [32]. The two amino protons, equivalent and easily exchangeable in the starting compound, became not equivalent and not easily exchangeable when the α-amino group coordinated the Pt ion. In addition, both proton and carbon signals of the CH-α (indicated as E) in **T1** and **T2** were markedly downfield shifted with respect to the corresponding signal in the starting amino acid (Table 1), indicating the presence of platinum in the proximity of this group. In the ^13^C NMR spectra of both complexes, a downfield shift from 168 to about 181 ppm of the carboxylic carbon F was observed, indicating the coordination of the carboxylic group to the metal center. Strong evidence of the presence of DMSO as a platinum ligand in both Pt complexes came from new proton and carbon signals in **T1** and **T2**, attributable to the methyl groups linked to the Pt-bound sulfur (downfield-shifted with respect to free DMSO), in agreement with chemical shifts reported in the literature for DMSO-containing Pt complexes [5,15].

Notably, in the explored conditions, only for **T1** and not for **T2,** the presence of two rotamers (doubling of some proton/carbon signals) was observed. Rotamers, which are generally reported for peptide nucleic acid (PNA) or some nucleoamino acid monomers [33,34], are due to a restricted rotation around the amide bond. Thus, it can be deduced that the rotation of the nucleobase around the methylene group occurred more rapidly for **T2** than for **T1** in the NMR time scale.

All the NMR data indicated that both complexes coordinate platinum through the α-NH_2_ and α-COOH groups, whereas the nucleobase, the methylenecarbonyl linker, and the amidic nitrogen were not involved in the metal center complexation. Thus, **T1** and **T2** can differ only for the relative position of the DMSO and chloride ligands. To confirm the exact position of these ligands in **T1** and **T2**, nuclear Overhauser effect spectroscopy (NOESY) experiments were also performed. A diagnostic cross peak between the proton of the amino group coordinating the platinum and the DMSO-methyl groups was found in **T2** NOESY spectrum but not in **T1** (Table S4 and Figure S9). Finally, molecular modeling analysis of the Pt complexes, by applying the MMFF94 energy minimization method [35,36], confirmed the observed NOESY correlations (Figures S10 and S11, realized using the MOLVIEW program [35]). Taken together, the obtained data concurred to define for **T1** and **T2** the structures reported in Scheme 1, whereas for the initially obtained complexes, we hypothesize the structures depicted in Figure S11 (bottom).

To definitively confirm the structures of **T1** and **T2**, as well as of their precursors, we tried to crystalize them in different ways to perform X-ray diffraction studies. Unfortunately, attempts to obtain good quality crystals were unsuccessful, and only decomposition products were observed.

### 2.3. UV-Vis Analysis of **T1** and **T2** Platinum Complexes in Aqueous Solution

Before analyzing the UV-vis behavior of **T1** and **T2** in aqueous solution, we first characterized the absorption spectral properties of the starting nucleoamino acid DAP(T)-OH. The UV spectrum of DAP(T)-OH, dissolved at 80 μM conc. in PBS (phosphate-buffered saline, 137 mM NaCl, 2.7 mM KCl, 10 mM Na_2_HPO_4_, 1.8 mM KH_2_PO_4_), showed an intense band at 268 nm, characteristic of the thymine nucleobase (*ε*_260_ = 8560 M^−1^cm^−1^) [37]. The UV signal was monitored over time up to 96 h. Apart from a very slight increase in the 268-nm band in the first 24 h, no significant spectral changes were observed over a longer monitoring time, indicating good stability of DAP(T)–OH in the tested buffer solution (Figure S12).

**Figure 2:**
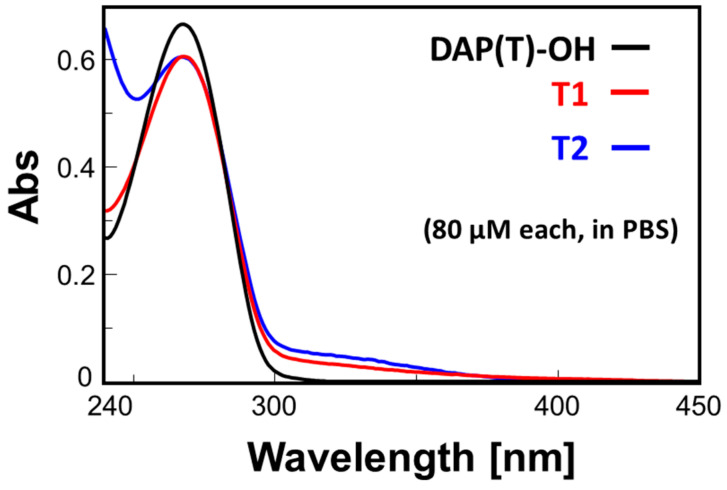
Overlapped ultraviolet-visible (UV-vis) spectra of the two platinum complexes (**T1** and **T2**) and the starting ligand DAP(T)–OH dissolved at 80 µM concentration in phosphate-buffered saline (PBS).

The UV-vis spectra of **T1** and **T2** complexes in PBS showed, in both cases, the main band relative to the thymine chromophore almost overlapped to that of DAP(T)-OH, further confirming that the nucleobase does not participate to the Pt coordination. This band, having a high extinction molar coefficient, probably submerged possible absorption bands relative to metal/ligands charge-transfer transitions. Indeed, only a weak band at about 330 nm, which proved the presence of the metal in the complexes, was detected (Figure 2).

The stability of the complexes was evaluated by monitoring their UV spectra in PBS over time for 48 h. In the first hour of monitoring, an increase in both the 268 and 330 nm bands occurred with a concomitant 1 nm red-shift of the maximum of the 268-nm band (Figures S13a and S14a). Then, after 1 h, the weak metal-to-ligand charge transfer (MLCT) band started to decrease, probably due to the exchange reactions between water and the labile ligands. The overall UV signal stabilized after 48 h (Figures S13b and S14b).

Overall, the variations in the UV-vis spectra of both metal complexes were barely detectable, resulting in a slight hyperchromic- and red-shift of the band at 268 nm and a small decrease in the 330 nm band (Figures S13c and S14c).

### 2.4. NMR Analysis of **T1** and **T2** Platinum Complexes in Aqueous Solution

Since only moderate changes were detected in the UV-vis spectra of **T1** and **T2** in aqueous solution during the time-course experiments, due to the strong absorption of the nucleobase with respect to the weak MLCT bands of the metal complexes, we followed their stability over time using another technique. In particular, by using ^1^H-NMR experiments, we observed that both the platinum complexes were stable for months in DMSO-d_6_. By adding 10% D_2_O to the DMSO-d_6_ solution of **T1** and **T2**, we evidenced the appearance of new signals and the intensity reduction of others, denoting that more than one species was formed from the original ones. However, with only 10% water, the complexes were still quite stable. Indeed, the new species started appearing after 8 h, and intact **T1** and **T2** were still present in the solution for almost 50% of their initial amount after 48 h (see Figure S15a for **T2**). Probably, the first exchanged ligand by water was chloride, followed (after at least 24 h) by DMSO, as evidenced by the appearance of the free DMSO signal (at 2.23 ppm). The hydrolysis of the Pt complexes was also monitored by dissolving them directly in D_2_O. Obviously, in this case, the process was faster, affording the complete exchange of the DMSO ligand after 48 h, and the detachment from the metal also of the DAP(T)–OH ligand after 96 h (see Figure S15b for **T2** complex). Even if monitoring **T1** behavior was complicated by the presence of the rotamers, which rendered the spectra interpretation more difficult, by following the appearance of the free DMSO signal in both the complexes dissolved in D_2_O, we observed a slightly faster exchange of this ligand for **T2** than for **T1** (Figure S16).

### 2.5. Antiproliferative Activity of the **T1** and **T2** Platinum Complexes

**Figure 3:**
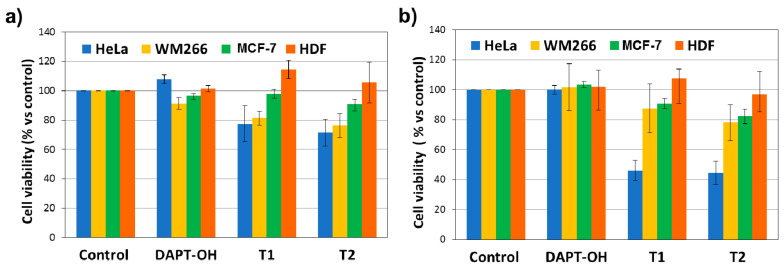
Cell viability assay on human cervical adenocarcinoma (HeLa), metastatic melanoma (WM266), breast adenocarcinoma (MCF-7), and human fibroblasts (HDF). Cells were incubated with **T1**, **T2,** or DAP(T)–OH at 25 (**a**) or 50 (**b**) μM concentration at 37 °C for 48 h. Cell viability was measured by using the 3-(4,5-dimethylthiazol-2-yl)-2,5-diphenyltetrazolium bromide (MTT) assay. The results are presented as the percentage of living cells with respect to the control (vehicle-treated cells) and are expressed as means ± SE of at least three independent experiments performed in triplicate.

The antiproliferative effects of **T1** and **T2** complexes were evaluated on three different tumor cell lines (i.e., HeLa, human cervical adenocarcinoma; WM266, metastatic melanoma; MCF-7, breast adenocarcinoma) and one healthy cellular model (HDF, human fibroblasts). Cells were treated with solutions of Pt(II) complexes and DAP(T)–OH freshly diluted in growth medium at 25 and 50 µM concentrations, and after 48 h, the 3-(4,5-dimethylthiazol-2-yl)-2,5-diphenyltetrazolium bromide (MTT) assay was performed. The percentage of living cells with respect to the control (cells treated only with the vehicle) for each compound is reported in Figure 3.

The results indicated that the complexes showed a good antiproliferative activity on HeLa cells with a cell viability reduction of 23% and 29%, at 25 µM, and 55% and 54% at 50 µM, respectively, for **T1** and **T2**. The target complexes were less active on WM266 and MCF-7 cells, reducing their viability by about 20% at the highest concentration tested (50 µM). In turn, the free ligand DAP(T)–OH did not affect the proliferation of all the tested cells, indicating that the activity found for **T1** and **T2** is essentially due to the presence of platinum.

However, **T1** and **T2** complexes proved to be less active than cisplatin (used as positive control) on the treated tumor cells, but, remarkably, more selective with respect to the healthy ones (Figure S17). In fact, **T1** and **T2** did not interfere with the HDF proliferation at the tested concentrations (Figure 3).

### 2.6. CD-Monitored Binding Experiments of the New Platinum Complexes with DNA Model Systems

Subsequently, the novel Pt(II) complexes **T1** and **T2,** as well as the nucleoamino acid DAP(T)–OH, were studied by CD spectroscopy in their interaction in solution with DNA sequences representing useful model systems [38,39]. In detail, we used a 12-mer random coil single strand (ODN1) containing a central box with two contiguous guanines (*GG* box), and three secondary structure-forming oligonucleotides (ODNs), i.e., two hairpin duplexes of 12 base-pairs (one with and the other without a central *GG* box), and a unimolecular G-quadruplex (G4) model with three G-quartets. The hairpin duplexes were formed by two 27-mer self-complementary single strands, classical models for B DNA duplexes (ds27: two tracts of the Dickerson sequence joined by a central three-thymine (TTT) loop [40,41]; and ds*GG*: two 12-mer tracts, one of which contained a *GG* box, joined by a central TTT loop), whereas the unimolecular G4 was derived from the 26-mer human telomere sequence (tel_26_), which can form in proper conditions G-quadruplex structures. The strong interest for G-quadruplexes is due to the formation of these non-canonical DNA structures in some regions of the human genome, especially in telomeres and in regulatory regions of some oncogenes [42,43,44].

All the CD-binding experiments were performed in a K^+^-rich buffered solution (100 mM KCl, 7 mM Na_2_HPO_4_/NaH_2_PO_4_, pH = 7.2) to mimic the intracellular physiological conditions. The ODNs used as DNA model systems were subjected to the annealing procedure taking the sequences in the selected buffer at high temperature (95 °C) for 5 min, and then leaving them to slowly cool to room temperature. This procedure allows structuring either the duplexes and G4 structures, as verified by the CD spectra (Figure S18). The conformation adopted by tel_26_ after annealing in the selected buffer was essentially a hybrid-2 type structure, as evidenced by the comparison of its CD spectrum in Figure S18d with those reported in the literature [45,46].

Since the contribution to the CD signal of the starting nucleoamino acid and the Pt complexes was negligible at the concentration used for the experiments (Figure S19), we monitored the CD profiles of the ODN systems before and after incubation with the target molecules (10 equiv.) dissolved in DMSO, at different time periods (0, 2, 24, and 48 h) after the addition. As a reference, we also monitored upon time the CD signal of the ODN solutions after the addition of pure DMSO (same volume as the one added in previous incubations with the compounds) without evidencing significant changes in the overall CD spectra.

**Figure 4:**
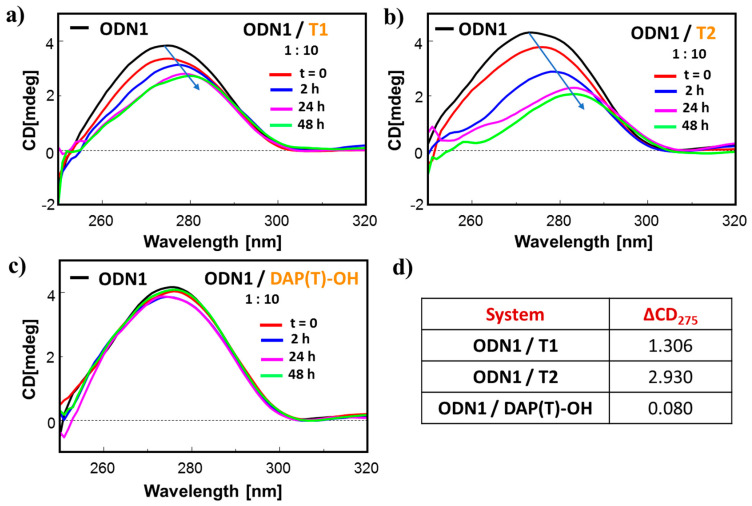
Overlapped circular dichroism (CD) spectra of oligonucleotide 1 (ODN1) (2 µM concentration), in the absence (black lines) and presence of **T1** (**a**), **T2** (**b**), and DAP(T)–OH (**c**) (20 µM each) at different times (0, 2, 24, 48 h) after the addition of the target molecules. (**d**) Differences in the CD signal at 275 nm between the CD spectra of ODN1 before and after 48 h incubation with each added compound. The experiments were carried out in 100 mM KCl, 7 mM Na_2_HPO_4_/NaH_2_PO_4_, pH = 7.2, at 20 °C (optical path = 1 cm).

As shown in Figure 4a,b, significant changes in the CD spectrum of ODN1 were observed when this was treated with **T1** and **T2**, evidencing that both complexes interacted with this single strand oligonucleotide. In contrast, the starting nucleoamino acid did not produce relevant CD variations on ODN1 (Figure 4c). In detail, in the presence of **T1**, the CD spectrum of ODN1 showed a decrease over time of the band at 275 nm accompanied by a red-shift of the band maximum reaching the highest effects 48 h after the complex addition (ΔCD_275_ = 1.3 mdeg, 5 nm red-shift). More marked changes in the CD band were observed when complex **T2** was added to the ODN1 solution. Upon time, the CD spectrum exhibited a dramatic decrease in the CD signal at 275 nm (ca 70%) accompanied by a 10 nm red-shift of the band maximum.

Similarly to ODN1, CD spectroscopy was used to evaluate the interaction of **T1**, **T2**, and DAP(T)–OH with the selected duplex DNA models. In these cases, small changes in the CD spectra of the duplexes were observed when **T1** and **T2** were added to the oligonucleotide solutions (Figure S20a,b,e,f), whereas no changes were recorded for the incubation with DAP(T)–OH (Figure S20c,g). In particular, with both the duplex systems, a reduction in the CD signal of about 1 mdeg was observed after incubation with **T1** (Figure S20a,d for ds27, and Figure S20e,h for ds*GG*). **T2** also produced a red-shift of the band maximum of the duplexes of ca. 2 nm, along with a CD intensity decrease, which, however, was more pronounced for the duplex containing the *GG* box compared to ds27 (Figure S20b,d for ds27 and Figure S20f,h for ds*GG*).

**Figure 5:**
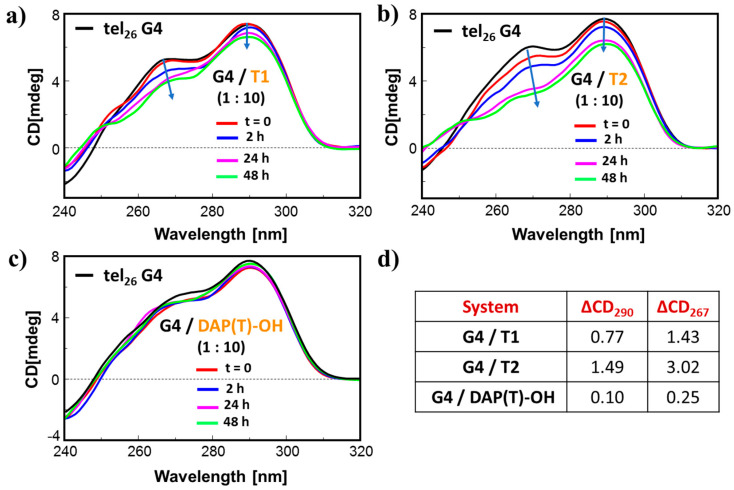
Overlapped CD spectra of the G-quadruplex (G4) tel_26_ (2 µM concentration), in the absence (black lines) and presence of **T1** (**a**), **T2** (**b**), and DAP(T)–OH (**c**) (20 µM each) at different times (0, 2, 24, 48 h) after the addition of the target molecules. (**d**) Differences in the CD signal at 290 and 267 nm between the CD spectra of the G4, before and after 48 h incubation with each added compound. The experiments were carried out in 100 mM KCl, 7 mM Na_2_HPO_4_/NaH_2_PO_4_, pH = 7.2, at 20 °C (optical path = 1 cm).

As far as the interactions with the G4 was concerned, only slight differences between the CD spectrum of tel_26_ in the absence and presence of the nucleoamino acid DAP(T)–OH were observed up to 48 h (Figure 5c). In contrast, after the addition of **T1** or **T2** to the G4 solution, both the main band at 290 nm and the shoulder at 267 nm significantly decreased over time, reaching, also in this case, the maximum effects after 48 h incubation (Figure 5a,b).

Taken together, the CD data suggested that DNA can be a possible target of the newly obtained compounds, similar to other well-known anticancer Pt complexes [6,7,12,21,23,47,48], with the DAP(T)-based Pt complexes inducing important conformational changes in G4 structures and single stranded ODNs upon binding, contrarily to the duplex structure (either containing or not the *GG* box).

To compare the ability of the complexes to bind the different DNA systems with that of cisplatin, the same CD experiments performed with **T1** and **T2** were also carried out with CDDP under identical experimental conditions. In agreement with our previous work [47], CDDP proved to significantly interact with the random-coil single strand containing a *GG* box (ODN1, Figure S21a) and the *GG*-containing duplex (ds*GG*, Figure S21c), whereas it was not able to perturb or disrupt the compact G4 structure of tel_26_ (Figure S21d) nor the duplex not-containing the *GG* box (Figure S21b). Furthermore, while CDDP evidenced a clear selectivity towards duplex structures containing a *GG* box (ds*GG* vs. ds27, Figure S21b,c), **T1** and **T2** did not efficiently interact with duplex structures, either containing or not a *GG*-box element (Figure S20b,c).

Finally, since the UV bands of **T1**, **T2**, and DAP(T)–OH significantly overlapped with those of the selected ODN systems, contrarily to CD analysis, UV data were not useful to interpret the binding behavior of these complexes towards different DNA structures.

### 2.7. PAGE-Monitored Binding Experiments of the New Platinum Complexes with DNA Model Systems

**Figure 6:**
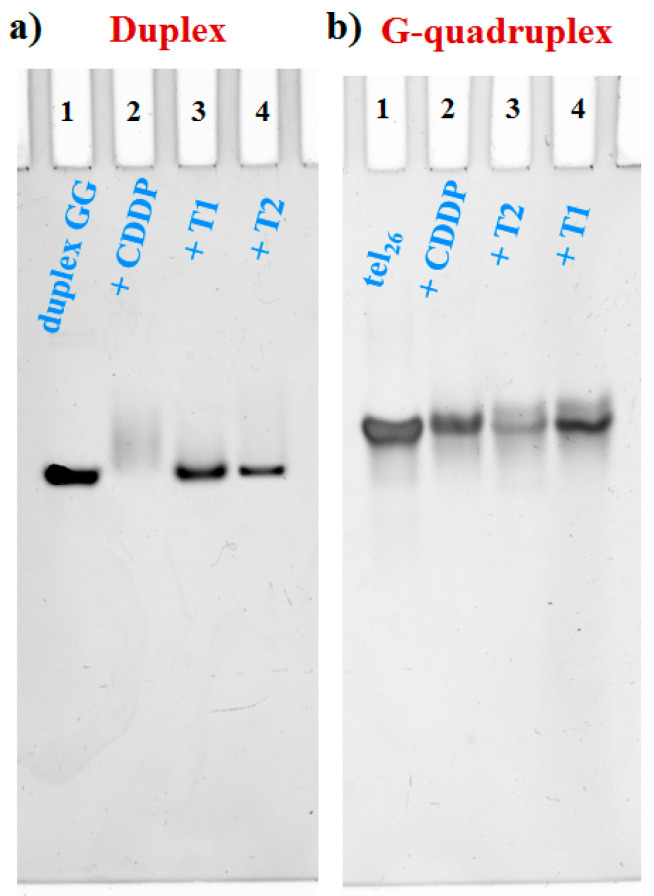
Representative 25% polyacrylamide gel electrophoresis (PAGE) under native conditions relative to the *GG*-duplex (**a**) and the G-quadruplex (**b**) DNA systems (2 µM concentration), alone (lanes 1) or incubated for 48 h with 10 equiv. of CDDP, **T1** and **T2** in 100 mM KCl, 7 mM Na_2_HPO_4_/NaH_2_PO_4_, pH = 7.2. Gel was run at 80 V, at r.t. for 3.3 h in TBE 1× buffer.

The structured DNA model systems that had evidenced, from the CD studies, the higher conformational variations upon binding with the Pt complexes, were selected for native polyacrylamide gel electrophoresis (PAGE) experiments to obtain qualitative data on DNA platination and compare the behavior of **T1** and **T2** with that of CDDP. In particular, the G4 tel_26_ and the duplex containing the *GG* box (ds*GG*) were incubated separately for 48 h with 10 equiv. of **T1**, **T2**, and CDDP at room temperature. Inspection of the gel, reported in Figure 6a, evidenced that DNA platination on the *GG*-duplex occurred efficiently by CDDP, as expected, and to a less extent by **T1** and **T2** (with **T2** > **T1**), whereas the G4 tel_26_ was platinated more by **T1** and **T2** (**T2** > **T1**) than by CDDP (Figure 6b). Thus, PAGE data were in perfect agreement with the CD results (Figure S20e,f, Figure 5a,b, and Figure S21c,d).

To exclude the contribution of DMSO in the appearance of additional, smeared DNA bands after ligand incubation, the oligonucleotides were also treated in the same conditions with equal amounts of this solvent without the ligands. In these experiments, no difference with the reference DNA bands was evidenced, demonstrating that the observed effects were not due to the solvent (Figure S22).

## 3. Materials and Methods

### 3.1. Reagents, Instruments, and General Methods

All the reagents and solvents were of the highest commercially available quality and were used as received. The coupling reactions were performed under an argon atmosphere, and all the reagents were previously co-evaporated with anhydrous CH_3_CN. Thin layer chromatography (TLC) analyses were carried out on silica gel plates from Macherey–Nagel (60, F254). Reaction products on TLC plates were visualized by 254 nm UV-light. DNA sequences were provided by Biomers.net GmbH (Ulm, Germany).

NMR spectra were recorded on Bruker Avance III HD 400 MHz and Varian Inova 500 MHz NMR spectrometers, as specified. All the chemical shifts (*δ*) in ^1^H- and ^13^C-NMR spectra are expressed in ppm with respect to the residual solvent (C*H*D_2_SOCD_3_, DMSO-d_5_, *δ* = 2.50 ppm, quintet) or tetramethylsilane (TMS) signals. All the coupling constants (*J*) are quoted in Hertz (Hz). The following abbreviations were used to explain the multiplicities: s = singlet; d = doublet; t = triplet; q = quartet; quin = quintet; m = multiplet; b = broad.

MALDI-TOF mass spectrometric analyses were performed on a TOF/TOF™ 5800 System in a positive mode, using 2,5-dihydroxybenzoic acid (DHB) or α-cyano-4-hydroxycinnamic acid (CHCA) as the matrices. For the deposition on the MALDI plate, the droplet spotting method was used. The sample (1 mg/mL in CH_3_CN/CH_3_OH or CH_3_CN/H_2_O, 7:3, *v*/*v*) was first deposited on the plate (0.5 µL) followed by matrix deposition (10 mg/mL in the same solvent mixture of the sample) and properly mixed before drying the components.

LC-ESI-MS analyses were performed on an Agilent 6230B TOF LC/MS instrument, equipped with a 1260 Infinity HPLC analytical system with binary pump coupled with a 6230 time of flight mass spectrometer as a detector and an Electrospray source; an Agilent ZORBAX C18 column (1.8 µm, 50 × 4.6 mm) for reverse-phase liquid chromatography were used. Stock solutions of the samples were prepared at 2 mg/mL concentration in HPLC grade CH_3_CN/H_2_O. The injections were carried out at a final concentration of 4 ng/μL in CH_3_CN/H_2_O, using a flow rate of 0.4 mL/min. Column elution was performed using H_2_O (A) (with or without 0.05% TFA, as specified) and CH_3_CN (B) as eluents. An isocratic elution of B in A for 3 min was followed by a gradient elution increasing B over 10 min. The ESI/TOF-MS was operated in positive ion mode (100–1500 *m*/*z* values).

The semipreparative purifications were carried out on a Hewlett Packard/Agilent 1100 series HPLC system, equipped with a UV detector, using a Phenomenex Luna C18 100 Å (5 μm, 4.6 × 150 mm) column. Gradient elution was carried out at 25 °C (monitoring at 254 nm) by using a gradient that started with buffer A (0.05% TFA in water) and applying buffer B (0.05% TFA in acetonitrile) with a flow rate of 1 mL min^−1^.

Samples were lyophilized in an FD4 Freeze Dryer (Heto Lab Equipment) for 16 h.

UV-vis measurements were performed on a JASCO V-550 UV-vis spectrophotometer equipped with Peltier Thermostat JASCO ETC-505T.

CD spectra were recorded with a Jasco J-715 spectropolarimeter equipped with a Peltier-type temperature control system (model PTC-348WI).

Energy-minimized 3D structure models (random low energy conformers) were obtained by using the 3D rendering engine JSmol and applying the MMFF94 energy minimization method [36] coded in MolView v2.4 (http://molview.org) software [35].

Acrylamide/bis-acrylamide (19:1) 40% solution, GelGreen Nucleic Acid Stain, and Tris-Borate- Ethylenediaminetetraacetic Acid (EDTA) (TBE) 10X were purchased from VWR. Ammonium persulfate (APS) and tetramethylethylenediamine (TEMED) were purchased from Sigma–Aldrich.

### 3.2. Synthesis of the DAP(T)–OH Nucleoaminoacid

#### 3.2.1. Fmoc-DAP(T)–OH, (S)-2-[(9*H*-fluoren-9-yl)methoxycarbonylamino]-3-{2-[5-methyl-2,4-dioxo-3,4-dihydropyrimidin-1(2*H*)-yl]acetamido} propanoic acid (**2**)

Fmoc-protected nucleoamino acid **3** (Scheme 1) was obtained as previously reported, with minor modifications [30]. Briefly, commercially available Fmoc/Boc-protected l-2,3-diaminopropanoic acid **1** (Fmoc-l-Dap(Boc)-OH: 51 mg, 0.120 mmol; Scheme 1) was treated with a 1:1 TFA/CH_2_Cl_2_ solution (2 mL), and the mixture was left under stirring at 45 °C for 1.5 h. Then, the solvent was removed in vacuo, and the crude was treated with cold diethyl ether. A white precipitate, recovered by filtration, was repeatedly washed with cold diethyl ether and finally dried in vacuo. This product corresponded to desired Fmoc-DAP-OH (**2**, 38 mg, 0.118 mmol, almost quantitative yield), as confirmed by TLC and NMR analysis [30]. Compound **2** was then dissolved in anhydrous DMF (1 mL), treated with DIEA (1 equiv., 0.118 mmol, 21 μL) and reacted with commercially available thymine acetic acid (2.2 equiv., 0.260 mmol, 48 mg), previously activated with HATU (2.0 equiv., 0.230 mmol, 89 mg) and DIEA (4.0 equiv., 0.470 mmol, 82 μL) in DMF (1 mL) for 3 min. After 3 h at room temperature, under magnetic stirring and argon atmosphere, the solvent was removed in vacuo. The crude material was treated with a 0.1% TFA aq. solution and filtered off to remove the aqueous solution from the white precipitate. This solid was resuspended in a mixture of H_2_O/CH_3_CN (7:3, *v*/*v*) containing 0.1% TFA and purified by semipreparative HPLC according to a gradient elution starting from H_2_O/CH_3_CN (both containing 0.1% TFA) = 78:22, and increasing the amount of CH_3_CN to 70% in 20 min. The desired product (**3**), whose identity was confirmed by TLC, MS, and NMR analysis [30], was obtained as a pure compound in good yield (46 mg, 0.094 mmol, 80% yield).

#### 3.2.2. DAP(T)–OH, (S)-2-amino-3-{2-[5-methyl-2,4-dioxo-3,4-dihydropyrimidin-1(2*H*)-yl]acetamido} propanoic acid (**4**)

Isolated product **3** (46 mg, 0.094 mmol) was dissolved in 2 mL of a 10% piperidine solution in DMF:CH_3_OH (9:1, *v*/*v*). After 10 min stirring at room temperature, TLC analysis indicated the complete removal of the Fmoc group. Then the solvent was removed under vacuum and the crude was resuspended with cold diethyl ether obtaining a white precipitate. This species was recovered after centrifugation, by discarding the supernatant, washing with cold diethyl ether (3 × 5 mL) and drying. Lyophilization from H_2_O (containing 0.1% TFA) allowed obtaining the desired compound **4** in almost quantitative yield (25 mg, 0.092 mmol): white powder. *Rf =* 0.1 (CH_2_Cl_2_/CH_3_OH, 8:2, *v*/*v*). Mass calculated for **4** (C_10_H_14_N_4_O_5_ = M): 270.10 u.m.a.; MALDI-TOF-MS (positive ions, Figure S1, up) *m*/*z* found: 271.00 [M + H]^+^, 293.13 [M + Na]^+^, 309.10 [M + K]^+^; ESI-MS (positive ions, Figure S1, bottom) *m*/*z* found: 271.14 [M + H]^+^, 292.98 [M + Na]^+^, 308.95 [M + K]^+^. ^1^H NMR (400 MHz, D_2_O; Figure S2, up): *δ* 7.44 (s, 1H, CH-6), 4.55 (bs, 2H, CH_2_-A), 3.93 (m, 1H, CHα-E), 3.87 (bd, *J* = 15.0 Hz, 1H, CH_2_-D_1_), 3.68 (bdd, 1H, *J* = 15.0, 5.9 CH_2_-D_2_), 1.90 (s, 3H, CH_3_-5). ^13^C NMR (100 MHz, D_2_O; Figure S2, bottom): *δ* 172.56 (COOH-F); 171.29 (C-B); 167.70 (C-4); 153.13 (C-2); 143.83 (CH-6); 111.85 (C-5); 55.69 (CH_2_-A); 51.18 (CH-E); 40.66 (CH_2_-D); 11.98 (CH_3_-5). ^1^H NMR (500 MHz, DMSO-d_6_; Figure S3, up): *δ* 8.35 (bt, 1H, NH-C); 7.41 (s, 1H, CH-6); 4.31 (AB system, 2H, CH_2_-A); 3.53 (bdt, *J* = 13.6, 4.5 Hz, 1H, CH_2_-D_1_); 3.33 (m, 1H, CH_2_-D_1_); 3.27 (m, 1H, CHα-E); 1.75 (s, 3H, CH_3_-5). ^13^C NMR (125 MHz, DMSO-d_6_; Figure S3, bottom): *δ* 168.82 (COOH-F), 167.77 (C-B), 164.36 (C-4), 151.05 (C-2), 142.31 (C-6), 108.01 (C-5), 54.04 (CH-E), 49.16 (CH_2_-A), 39.62 (CH_2_-D), 11.87 (CH_3_-5). UV-vis spectrum of **4** (Figure 2): band maximum at 268 nm (*ε*_260_ = 8700 M^−1^cm^−1^).

### 3.3. Platination Reaction of DAP(T)–OH

The nucleoamino acid derivative DAP(T)–OH (**4**, 25 mg, 0.092 mmol) was suspended in CH_3_OH (1.35 mL). In parallel, K_2_PtCl_4_ (1 equiv., 10.58 mg, 0.092 mmol) was dissolved in the minimum amount of H_2_O (150 μL). In 1 h, the amino acid solution was slowly added to the aqueous solution of K_2_PtCl_4_ (final solvents ratio: CH_3_OH/H_2_O, 9:1, *v*/*v*). The reaction mixture was kept under stirring in the dark and at room temperature for 6 h until the formation of an almost clear yellow solution from the initial dark orange one, with the concomitant disappearance of the DAP(T)–OH white suspension. Subsequently, the solvent was removed under vacuum at 30 °C, and the reaction crude was dried for 1 h at the oil pump to remove traces of H_2_O. Then, the crude mixture was sequentially resuspended after sonication in various solvents (dichloromethane, chloroform, and acetone) and centrifuged, affording, in all cases, a dark-yellow precipitate and a colorless supernatant. This solution, analyzed by TLC, did not show UV-vis bands and was consequently discarded. In contrast, treatment of the precipitate with CH_3_OH gave a pale-yellow solution and a dark-yellow precipitate. Thus, a series of centrifugations and washings of the precipitate with CH_3_OH (until the supernatant was completely colorless) was performed, and the combined yellow methanol solutions (ca. 6 × 5 mL) were taken to dryness. Then, the dark-yellow solid was resuspended by sonication with H_2_O obtaining a yellow supernatant and a white precipitate. After centrifugations and washings of the precipitate with H_2_O (until the supernatant was completely colorless), the aqueous solutions (ca. 3 × 5 mL) were combined, and immediately frozen and lyophilized to remove water from the complex. The white precipitate was then discarded, and the two obtained yellow components (yellow powders) were separately analyzed. LC-ESI-MS analysis of the two Pt complexes was performed as follows: the complexes were dissolved in H_2_O/CH_3_CN (9:1, *v*/*v*), injected on a C18 column, and eluted in gradient mode (3 min isocratic elution with 5% CH_3_CN in H_2_O, then gradient to 50% CH_3_CN in 10 min; *t*_R_ = 0.94 and 0.89 min, for methanol- and water-recovered Pt complexes, respectively; see Figure S4a).

Since the most common NMR organic solvents (CDCl_3_, CD_3_OD, CD_3_COCD_3_) did not solubilize the recovered Pt complexes at a proper concentration for a complete NMR characterization, we used DMSO for their solubilization. The newly obtained DAP(T)-based complexes were named **T1** and **T2** (originated from the DMSO-exchange reaction of the Pt complex recovered in CH_3_OH and H_2_O, respectively). The purity of the obtained complexes was checked by LC-ESI-MS, which evidenced the presence of a single peak for each compound (Figure S5a). For the LC-ESI-MS characterization, **T1** and **T2** were dissolved in H_2_O/CH_3_CN (9:1, *v*/*v*), injected on a C18 column, and eluted as follows: 3 min isocratic elution with 10% CH_3_CN in H_2_O, then gradient to 95% CH_3_CN in 10 min (*t*_R_ = 0.94 and 0.84 min, for **T1** and **T2**, respectively).

#### 3.3.1. **T1** Characterization

Mass calculated for **T1** (C_12_H_20_ClN_4_O_6_Pt = M): 578.23 u.m.a.; LC-ESI-MS (positive ions; Figure S5a, left) *m*/*z* found: 579.10 [M + H]^+^, 601.08 [M + Na]^+^, 617.06 [M + K]^+^ (chromatographic peak at 0.94 min). ^1^H NMR (400 MHz, DMSO-d_6_; Figure S6): *δ* 11.29/11.28 (s, two rotamers, NH-3/3′), 8.58/8.48 (bt, two rotamers, *J* = 5.7/5.6, NH-C/C’), 7.44/7.43 (s, two rotamers, H-6/6′), 6.29 (bs, NH_2_-G_1_), 5.68 (bs, NH_2_-G_2_), 4.35 (AB system, CH_2_-A), 3.90 (bm, CH_2_-E), 3.64 (bdt, *J* = 14.53, 5.06 Hz, CH_2_-D_1_), 3.59-3.26 (overlapped signals; in decreasing ppm order: CH_2_-D_1_′, CH_2_-E′, CH_2_-D_2_/D_2_′), 3.16 (bs, CH_3_-DMSO-Pt), 1.75 (s, CH_3_-5). ^13^C NMR (125 MHz, DMSO-d_6_; Figure S6): δ 181.33 (C-F), 168.86/168.31 (C-B/B’), 164.44/164.42 (C-4/4′), 151.05 (C-2), 142.24 (C-6), 108.10/107.98 (C-5/5′), 56.78 (CH-E), 52.40 (CH-E′), 49.22 (CH_2_-A), 49.02 (*C*H_3_-DMSO-Pt), 41.66 (CH_2_-D’), 38.58 (CH_2_-D), 11.93/11.91 (CH_3_-5/5′).

#### 3.3.2. **T2** Characterization

Mass calculated for **T2** (C_12_H_20_ClN_4_O_6_Pt = M): 578.23 u.m.a.; LC-ESI-MS (positive ions; Figure S5a, right) *m*/*z* found: 578.91 [M + H]^+^, 601.15 [M + Na]^+^, 617.24 [M + K]^+^ (chromatographic peak at 0.84 min). ^1^H NMR (400 MHz, DMSO-d_6_; Figure S7): *δ* 11.30 (s, 1H, NH-3), 8.43 (bs, 1H, NH-C), 7.43 (s, 1H, H-6); 6.25 (bs, 1H, NH_2_-G_1_), 5.63 (bs, 1H, NH_2_-G_2_), 4.34 (s, 2H, CH_2_-A), 3.65-3.27 (overlapped signals; in decreasing ppm order: CH_2_-D_1_, CH_2_-E, CH_2_-D_2_), 3.16 (bs, CH_3_-DMSO-Pt), 1.75 (s, 3H, CH_3_-5). ^13^C NMR (100 MHz, DMSO-d_6_; Figure S8): *δ* 181.38 (C-F), 168.44 (C-B), 164.47 (C-4), 151.07 (C-2), 142.23 (C-6), 108.16 (C-5), 56.90 (CH-E), 49.27 (CH_2_-A), 48.61 (*C*H_3_-DMSO-Pt), 41.69 (CH_2_-D), 11.94 (CH_3_-5).

Several attempts to obtain also MALDI-TOF spectra of the two complexes were performed using CHCA and DHB as matrixes and various laser powers, obtaining, in all cases, many adducts with the matrix and degradation products.

Based on their chemical structures (as deduced by NMR and mass data), the synthesis yields of **T1** and **T2** from the complexation reaction of DAP(T)–OH with K_2_PtCl_4_ followed by DMSO-exchange, were 35 and 23%, respectively.

Attempts to obtain crystals of the initial complexes, as well as of **T1** and **T2**, suitable for X-ray crystallography, failed, giving only degradation products (white crystals).

### 3.4. MTT Assay

#### 3.4.1. Cell Cultures

Human adenocarcinoma (HeLa), human metastatic melanoma (WM266), breast adenocarcinoma (MCF-7) cell lines were purchased from ATCC (U.S.). Normal human dermal fibroblasts (HDF) were kindly provided by Dr. A. Tito Arterra, Biosciences (Napoli, Italy). HeLa, MCF-7, and HDF were grown in Dulbecco’s Modified Eagle’s Medium (DMEM) supplemented with 10% fetal bovine serum (FBS), 1% glutamine, 100 U/mL penicillin, and 100 µg/mL streptomycin (Euroclone, Milano, Italy). WM266 were grown in RPMI, as previously described [38,39,49]. Cells were maintained in humidified air containing 5% CO_2_ at 37 °C.

#### 3.4.2. Cell Proliferation Experiments

Cells were plated at a density of 1200 cells/well for HeLa, 2000 for WM266, MCF-7, and HDF in 96-well microplates (Corning) and incubated at 37 °C. After 24 h, cells were treated for 48 h with the compounds previously solubilized. In detail, the Pt complexes and DAP(T)–OH were dissolved in DMSO at a 10 mM concentration, while cisplatin was solubilized in an aqueous solution of DMSO (10%)/NaCl (0.9%), at a 2 mM concentration [50], then diluted with growth medium until the final solution contained no more than 0.5% DMSO, well-tolerated by in vitro cell cultures. Cell proliferation was determined by using the 3-(4,5-dimethylthiazol-2-yl)-2,5-diphenyltetrazolium bromide assay (MTT, Sigma Aldrich, Milano, Italy) after 48 h incubation [51]. Plates were then analyzed using a microplate reader (Enspire, Perkin Elmer, Waltham, MA, USA) at 570 nm. The results are reported as the percentage of living cells with respect to the control (vehicle-treated cells) and are expressed as means ± SE of at least three independent experiments performed in triplicate.

### 3.5. UV-Vis Experiments

The UV spectra were recorded using a scanning speed of 100 nm/min and a 2.0 nm bandwidth with the appropriate baseline subtraction. A quartz cuvette with a path length of 1 cm was used (3.5 mL internal volume). The UV-vis spectra of the compounds were recorded in PBS (137 mM NaCl, 2.7 mM KCl, 10 mM Na_2_HPO_4_, 1.8 mM KH_2_PO_4_) over time in the range of 220 to 450 nm to study their stability. The solutions analyzed were 80 μM in PBS and were obtained after dilution from the original stock solution (7 mM in DMSO).

### 3.6. CD Experiments

The CD spectra were recorded at 20 °C in a quartz cuvette with a path-length of 1 cm using the following parameters: spectral window 240–320 nm, response 1 s, scanning speed 100 nm/min, bandwidth 2.0 nm. All the spectra were averaged over 3 scans and corrected by subtraction of the background scan with buffer. For all the experiments, oligonucleotides were dissolved at 2 μM concentration, stock solutions of ligands [**T1**, **T2**, and DAP(T)–OH] were prepared at 7 mM conc. in DMSO, whereas stock CDDP solution was obtained at a 7 mM concentration in an aqueous solution of DMSO (10%)/NaCl (0.9%) [50]. A buffer solution of 7 mM Na_2_HPO_4_/NaH_2_PO_4_, 100 mM KCl (pH 7.2) was used for the studies on the interaction between the DNA model systems and the platinum-based complexes.

The tested DNA sequences were the following (*GG* boxes are underlined and in italic):

ODN1 = CCTCT*GG*TCTCC (single strand model);

ds27 = CGCGAATTCGCG TTT CGCGAATTCGCG (duplex model not-containing a *GG* box);

ds*GG* = CATGT*GG*TTCTG TTT CAGAACCACATG (duplex model containing one *GG* box);

tel_26_ = TTA*GGG*TTA*GGG*TTA*GGG*TTA*GGG*TT (G-quadruplex model).

### 3.7. PAGE Experiments

Annealed oligonucleotides samples, dissolved at 2 μM concentration in 7 mM Na_2_HPO_4_/NaH_2_PO_4_, 100 mM KCl (pH 7.2) were incubated with 10 equiv. of each platinum complex. After 48 h incubation at room temperature (r.t.), the samples, including the reference DNA, were loaded on 25% polyacrylamide gels in TBE 1× as running buffer. All the mixtures were supplemented with 5% glycerol just before loading and then run, under native conditions, at 80 V at r.t. for 3.3 h. Gels were stained with a GelGreen solution (supplemented with 0.1 M NaCl) for 30 min and finally visualized with a UV transilluminator (BioRad ChemiDoc XRS, Milano, Italy). Each experiment was performed in duplicate.

## 4. Conclusions

In this work, the nucleoamino acid DAP(T)–OH, derived by the coupling of l-2,3-diaminopropanoic acid with a thymine acetic acid, was synthesized and characterized via mass spectrometry and NMR spectroscopy and used for the reaction with a platinum(II) salt to obtain novel Pt(II) complexes. Two new platinum-based complexes, named **T1** and **T2**, were obtained and characterized by various techniques. These compounds include in their structures one nucleoamino acid unit [DAP(T)–OH], a platinum(II) ion, a chloride ion, and a DMSO molecule. The identity of the new metal complexes was ascertained by NMR spectroscopy (^1^H and ^13^C, COSY, HSQC, HMBC, and NOESY experiments) and mass spectrometry (LC-ESI-MS) techniques.

The cytotoxicity of **T1** and **T2**, as well as of DAP(T)–OH used as a reference, was tested, in preliminary assays, on a panel of human cancer (HeLa, WM266, MCF-7) and normal (HDF) cells using the MTT assay. CDDP was also tested as a positive control. Both the Pt complexes showed good antiproliferative activity on HeLa cells. Indeed, **T1** and **T2** at 50 µM concentration reduced HeLa cell viability more than 50% after 48 h incubation, whereas, at the same tested concentration, they were less active on WM266 and MCF-7, suggesting a selectivity of action towards the specific tumor cells that is an important feature in cancer therapy. It is worth noting that, although **T1** and **T2** complexes resulted less effective in blocking cell proliferation than cisplatin, they were more selective towards cancer cells than healthy ones, as demonstrated by comparing the activity on HeLa cells with that observed on HDF ones, on which the complexes were essentially inactive even at the highest concentration tested. Interestingly, the free ligand DAP(T)–OH did not affect all the tested cells indicating that the observed bioactivity is essentially driven by the presence of the platinum center.

We also hypothesized that the nucleobase could contribute to the bioactivity of the Pt complexes by both protecting the Pt center from premature deactivation and increasing the cellular uptake of the complexes, plausibly thanks to specific nucleobase transporters, thus giving a synergistic effect together with the presence of the metal. Furthermore, generally, single nucleobases do not show relevant biological activity (nor significantly interact with DNA). In addition, as previously reported [21], the Pt complex with DAP-OH as ligand did not evidence significant antiproliferative activity in the same experimental conditions, showing a 50% inhibition of HeLa cells proliferation only at 220 μM concentration, whereas **T1** and **T2** seemed to be about 4-times more active on the same cancer cells.

Once the antiproliferative effects of the platinum-based complexes were assessed, to explore if their activity could be correlated with their DNA-binding ability, **T1** and **T2**, as well as DAP(T)–OH and cisplatin, used as reference compounds, were studied using CD spectroscopy. Their interaction in solution was investigated with four DNA model systems, i.e., a random coil single strand, two B form-duplexes, one with and the other without a *GG* box, and a G-quadruplex structure. From CD binding experiments, it emerged that DNA can be a possible target of these brand new compounds, similar to other reported bioactive Pt complexes (as well as transition metal complexes, such as ruthenium complexes) [6,7,12,15,21,23,47,48,52,53]. In particular, we observed significant changes only in the CD spectrum of the single strand and G4 when **T1** and **T2** were added to each DNA system, evidencing that both the complexes interacted with these oligonucleotides. A more marked interaction seemed to occur in the case of **T2** with respect to **T1**. On the contrary, the interaction of the starting nucleoamino acid with all the tested DNA systems was almost null, in line with the absence of specific antiproliferative effects observed in cell assays. Thus, CD data were in agreement with the observed biological activity for all the compounds. Furthermore, comparing the DNA-binding behavior of the new Pt complexes with that of cisplatin, it emerged that, while CDDP efficiently interacted with duplex structures containing a *GG* box not being able to perturb or disrupt the compact structure of the G4, **T1** and **T2** resulted good G4-binders as revealed by both CD- and PAGE-monitored binding experiments.

The moderate cytotoxicity combined with the selectivity of the Pt(II) complexes obtained and described in the present work, can be of pharmaceutical interest in multiple drug therapy. In addition, the synthesis and characterization of DAP-based nucleoamino acids also containing the other nucleobases, i.e., DAP(C)-OH, DAP(A)-OH, and DAP(G)-OH, are ongoing in our laboratories to use these nucleamino acid scaffolds as building blocks for the preparation of novel Pt complexes endowed with increased activity and selectivity, identifying the most promising analogs of the series.

## Figures and Tables

**Table 1:** Nuclear magnetic resonance (NMR)-chemical shift assignments for **T1** and **T2** metal complexes compared to those of the starting nucleoamino acid DAP(T)–OH (spectra recorded in dimethyl sulfoxide-d_6_ (DMSO-d_6_). Chemical shift values in blue for **T1** and **T2** indicate signals very similar to those of the starting ligand, whereas those in red are the most diverging ones; green-colored chemical shift values evidence proton and carbon signals of **T1** and **T2** not present in the starting nucleoamino acid.

^1^H-NMR				^13^C-NMR			
ppm	DAP(T)–OH	T1	T2	ppm	DAP(T)–OH	T1	T2
**C*H*_3_-5(T)**	1.75	1.75	1.75	***C*H_3_-5(T)**	11.87	11.93/11.91	11.94
**H-3 (T)**	11.25	11.29/11.28	11.30	**C-2 (T)**	151.05	151.05	151.07
**H-6 (T)**	7.41	7.44/7.43	7.43	**C-4 (T)**	164.36	164.44/164.42	164.47
**A**	4.31	4.35	4.34	**C-5 (T)**	108.01	108.10/107.98	108.16
**C**	8.35	8.58/8.48	8.43	**C-6 (T)**	142.31	142.24	142.23
**D_1_**	3.53	3.64/3.56	3.55	**A**	49.16	49.22	49.27
**D_2_**	3.33	3.49/3.28	3.29	**B**	167.77	168.86/168.31	168.44
**E**	3.27	3.90/3.52	3.51	**D**	39.62	41.66/38.58	41.69
**G_1_**	-	6.29	6.25	**E**	54.04	56.78/52.40	56.90
**G_2_**	-	5.68	5.63	**F**	168.82	181.33	181.38
**C*H*_3 (DMSO-Pt)_**		3.16	3.16	***C*H_3 (DMSO-Pt)_**		49.02	48.61

## Supplementary Materials

The following are available online at https://www.mdpi.com/1424-8247/13/10/284/s1.

## Abbreviations

A (adenine), APS (ammonium persulfate), B (nucleobase), Boc (tert-butoxycarbonyl), C (cytosine), CD (circular dichroism), CDDP (cis-diaminedichloro-platinum), CHCA (α-cyano-4-hydroxycinnamic acid), COSY (correlation spectroscopy), DAP (2,3-diaminopropanoic acid), DHB (2,5-dihydroxybenzoic acid), DIEA (N,N-diisopropylethylamine), DMEM (Dulbecco’s Modified Eagle’s Medium), DMF (N,N-dimethylformamide), DMSO (dimethyl sulfoxide), EDTA (Ethylenediaminetetraacetic Acid), ESI (electrospray ionization), FBS (fetal serum bovine), Fmoc (9-fluorenylmethoxycarbonyl), G (Guanine), G4 (G-quadruplex), HATU (1-[bis(dimethylamino)methylene]-1H-1,2,3-triazolo[4,5-b]pyridinium 3-oxid hexafluorophosphate), HDF (human dermal fibroblast), HeLa (cervical cancer cells), HMBC (heteronuclear multiple-bond correlation), HPLC (high-performance liquid-chromatography), HSQC (heteronuclear single quantum correlation), LC (liquid chromatography), MALDI (matrix-assisted laser desorption ionization), MCF-7 (human breast adenocarcinoma cell line), MS (mass spectrometry), MTT (3-(4,5-dimethylthiazol-2-yl)-2,5-diphenyltetrazolium bromide), NMR (nuclear magnetic resonance), NOESY (nuclear overhauser effect spectroscopy), ODN (oligodeoxyribonucleotide), PAGE (polyacrylamide gel electrophoresis), PBS (phosphate-buffered saline), PNA (peptide nucleic acid), ppm (parts per million), R_f_ (retention factor), r.t. (room temperature), TBE (Tris-Borate-EDTA), TEMED (tetramethylethylenediamine), TFA (trifluoroacetic acid), TOC (Thin layer chromatography), TOF (time of flight), *t*_R_ (retention time), WM (Wistar melanoma cell lines).
