# Supplementary material for: Synthesis, Antiproliferative Activity, and DNA Binding Studies of Nucleoamino Acid-Containing Pt(II) Complexes
Source: Pharmaceuticals (Basel). 2020 Sep 30;13(10):284. doi: 10.3390/ph13100284 (PMC7600948; doi:10.3390/ph13100284)
Supplement: Supplementary file 1 [file pharmaceuticals-13-00284-s001.pdf]

Supporting Information of the manuscript

Synthesis, antiproliferative activity and DNA binding studies of nucleoamino acid-containing Pt(II) complexes

Claudia Riccardi,[a§] Domenica Capasso,[b§] Angela Coppola,[a] Chiara Platella,[a]
Daniela Montesarchio,[a] Sonia Di Gaetano,[c] Giovanni N. Roviello,[c] Domenica Musumeci[a,c,*]

[a] *Department of Chemical Sciences, University of Napoli Federico II, 80126, Napoli, Italy;*
[b] *CESTEV, University of Naples Federico II, 80145 Naples, Italy;*
[c] *CNR, Institute of Biostructure and Bioimaging, 80134 Napoli, Italy*

*Correspondence to:
Domenica Musumeci, *Department of Chemical Sciences, University of Napoli Federico II, Via Cintia, 21, I-80126 Napoli, Italy*
E-mail address: domenica.musumeci@unina.it
Phone: +39-081-674143;

§ These authors contributed equally to this work

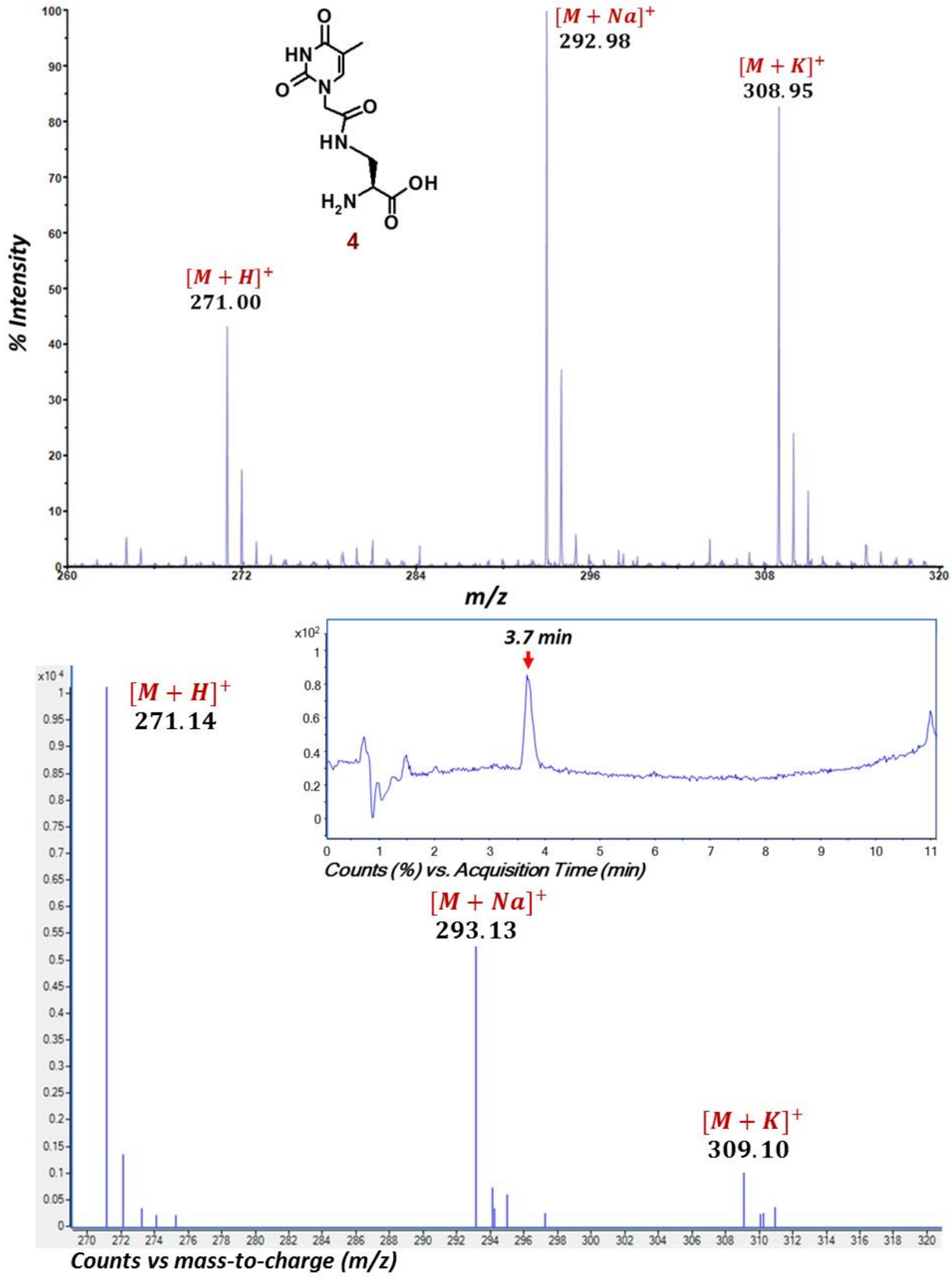

Figure S1: DAP(T)-OH MS characterization. MALDI-TOF mass spectrum of **4** using α-cyano-4-hydroxycinnamic acid (CHCA) as matrix (up); ESI mass spectrum from LC-ESI-MS analysis of **4** dissolved in H_2O/CH_3CN (9:1, v/v), injected on a ZORBAX C18 column (1.8 μm, 50 x 4.6 mm) and eluted as follows: 5 min isocratic elution with 10% CH_3CN in H_2O (0.05 % TFA), then gradient to 95 % CH_3CN in 10 min. TIC chromatogram of **4** is reported in the inset (t_R = 3.7 min) (bottom).

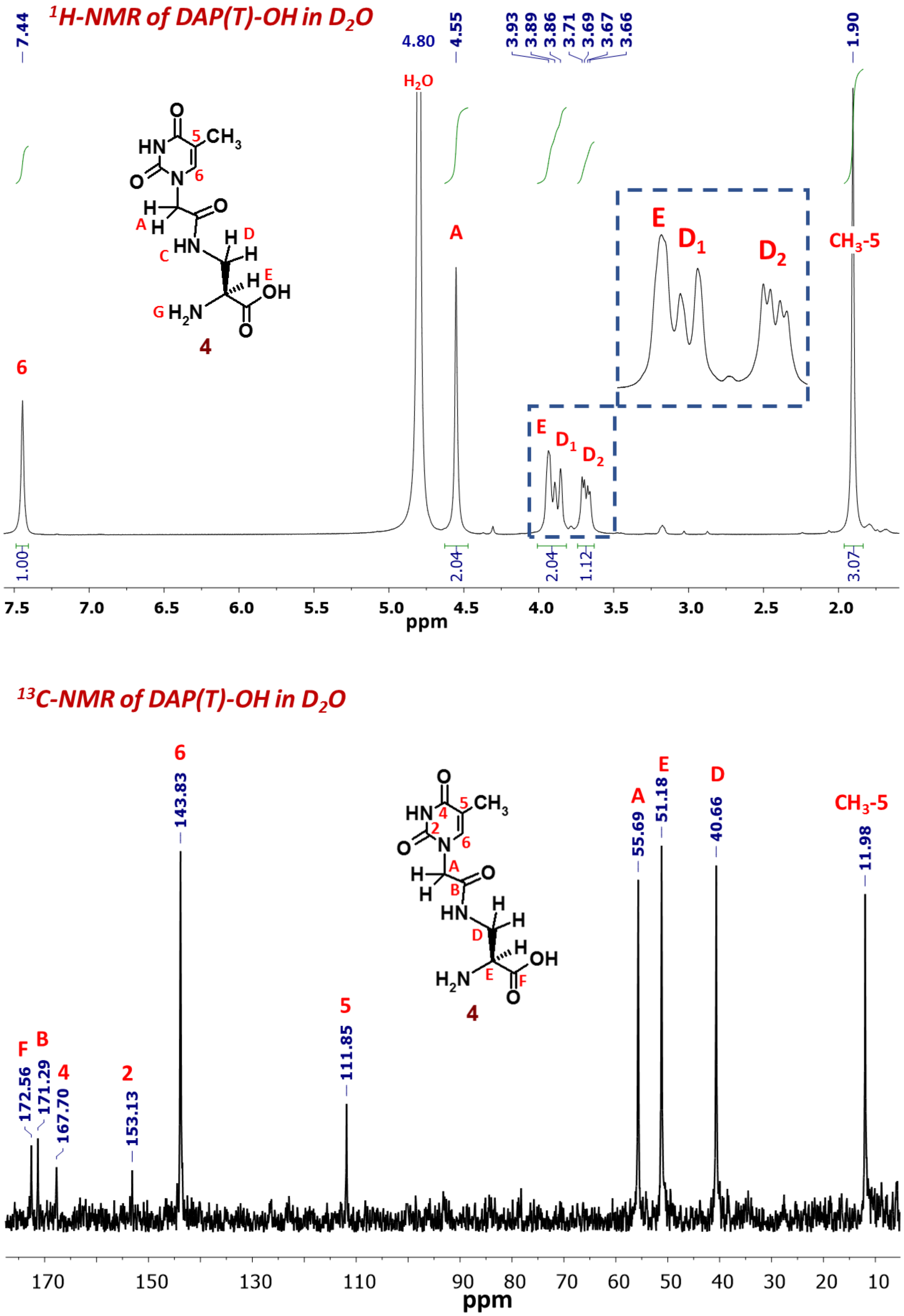

Figure S2: ^{1}H- (400 MHz) and ^{13}C-NMR (100 MHz) spectra of DAP(T)-OH in D_2O.

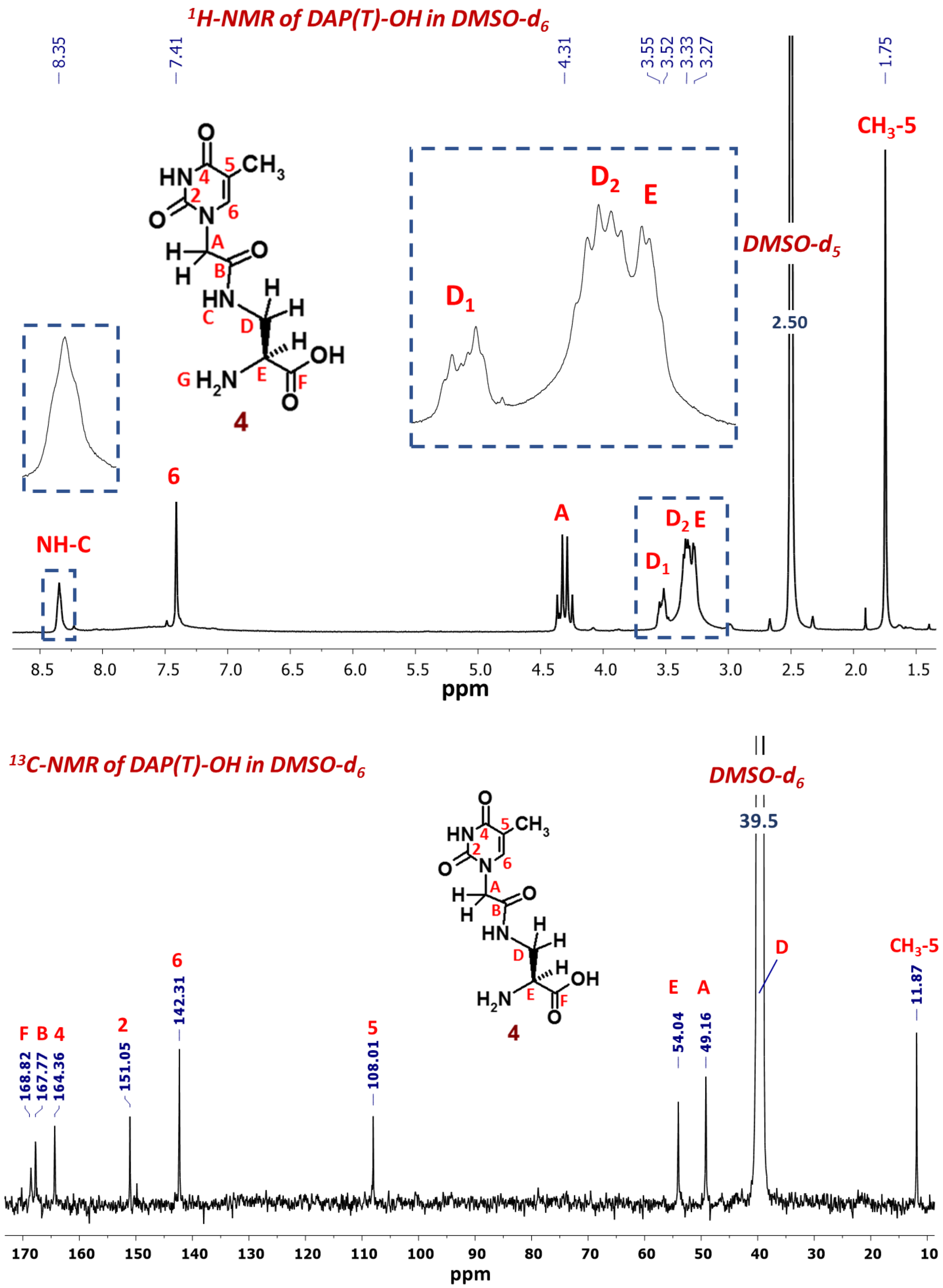

Figure S3: ^{1}H- (500 MHz) and ^{13}C-NMR (125 MHz) spectra of DAP(T)-OH in DMSO-d_6.

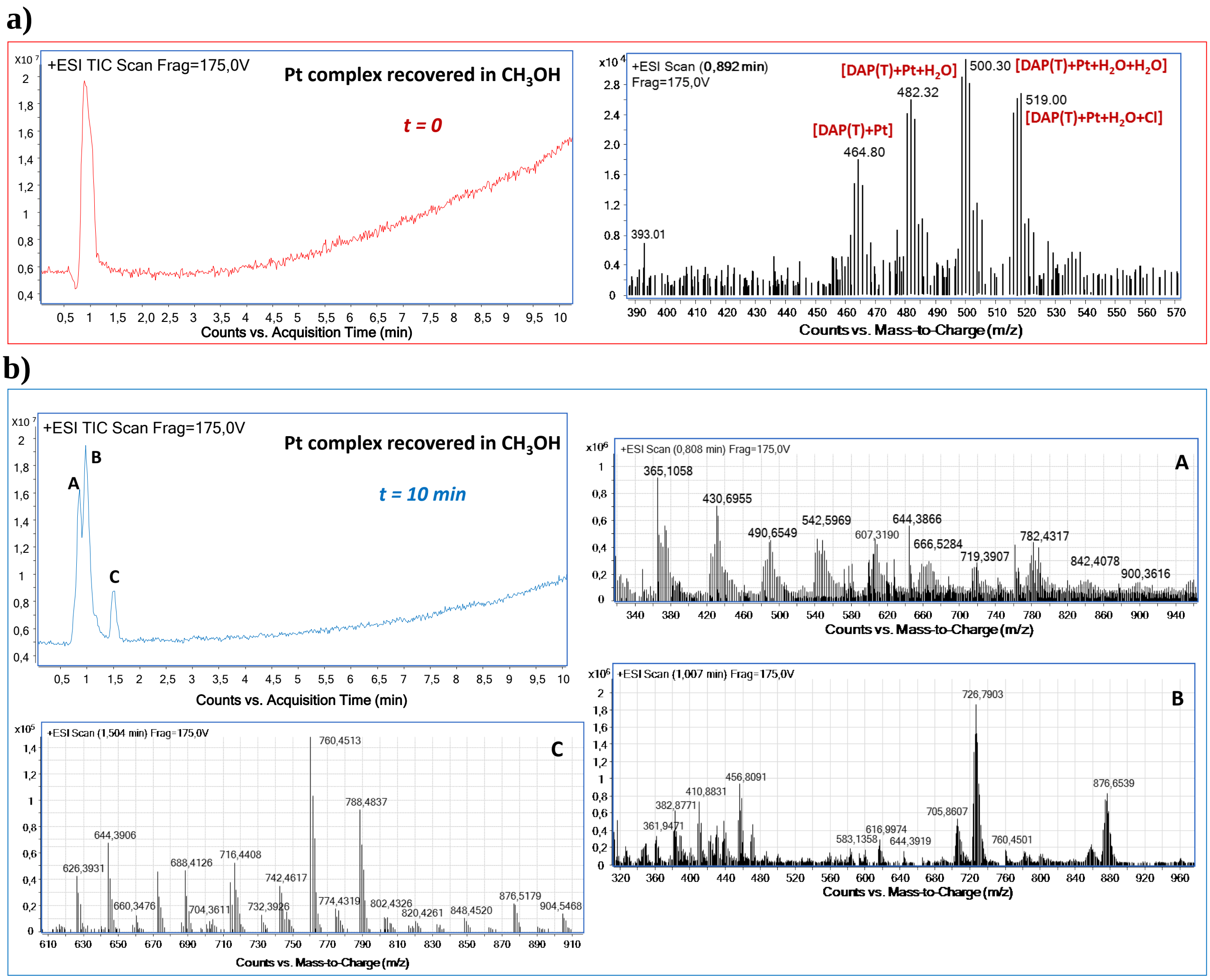

Figure S4: a),b) LC-ESI-MS analysis of the methanol-recovered Pt complex, dissolved in H_2O/CH_3CN (9:1, v/v), injected on a ZORBAX C18 column (1.8 µm, 50 x 4.6 mm) and eluted as follows: 3 min isocratic elution with 5 % CH_3CN in H_2O, then gradient to 50 % CH_3CN in 10 min. **a)** Pt complex immediately injected (t = 0) after its dissolution (single peak at t_R = 0.89 min); **b)** Pt complex injected 10 min after its dissolution (three peaks at t_R = 0.81, 1.01, 1.50 min).

a)

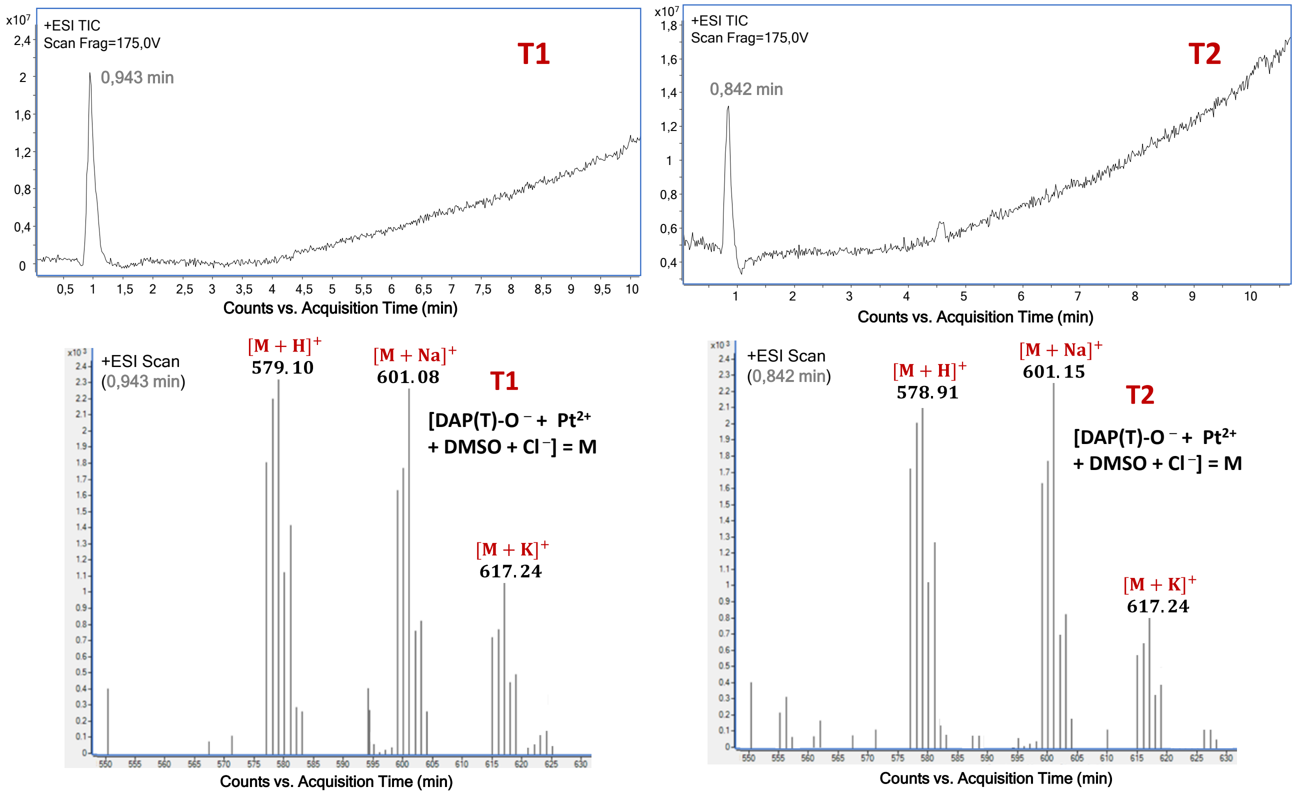

b)

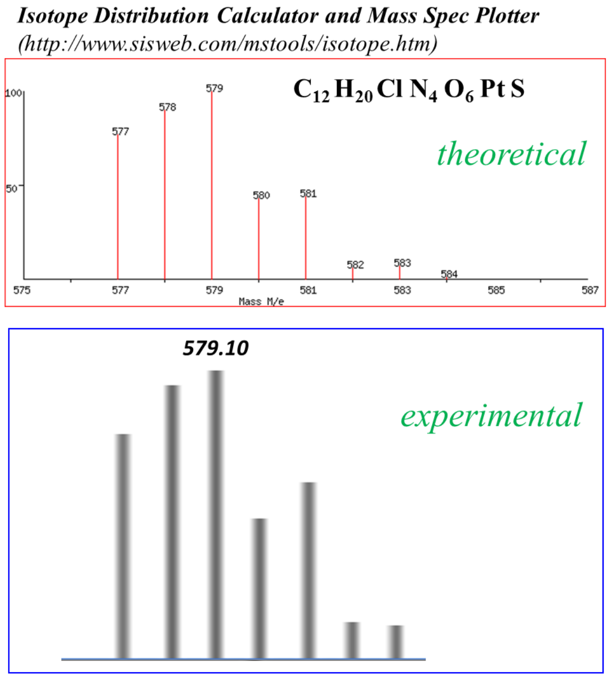

Figure S5: a) LC-ESI-MS analysis of **T1** (left) and **T2** (right) complexes dissolved in H_2O/CH_3CN (9:1, v/v), injected on a ZORBAX C18 column (1.8 μm, 50 x 4.6 mm) and eluted as follows: 3 min isocratic elution with 10 % CH_3CN in H_2O, then gradient to 95 % CH_3CN in 10 min (t_R = 0.94 and 0.84 min, for **T1** and **T2**, respectively); **b)** Comparison between theoretical and experimental mass isotope distribution of the peak at 579 m/z corresponding to the $[M-H]^+$ species of the complex **T1**.

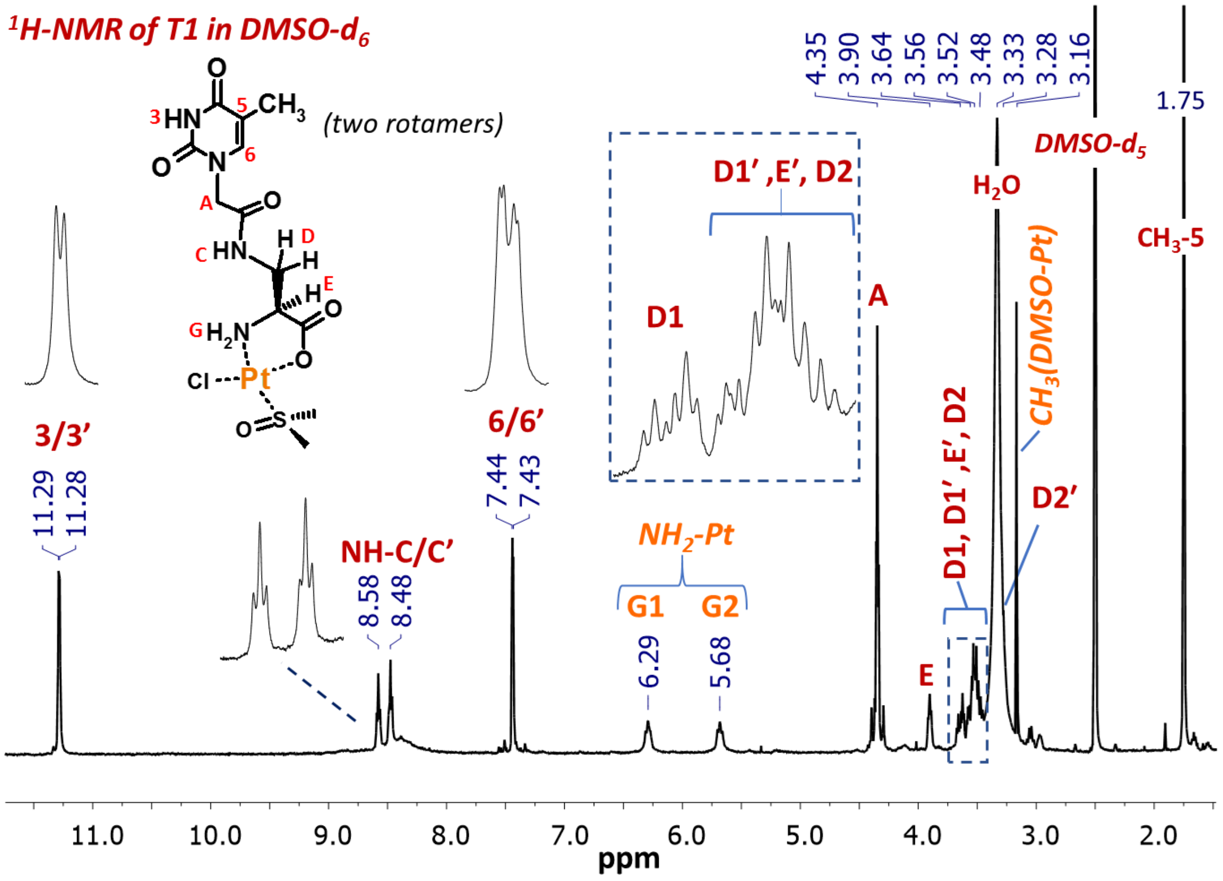

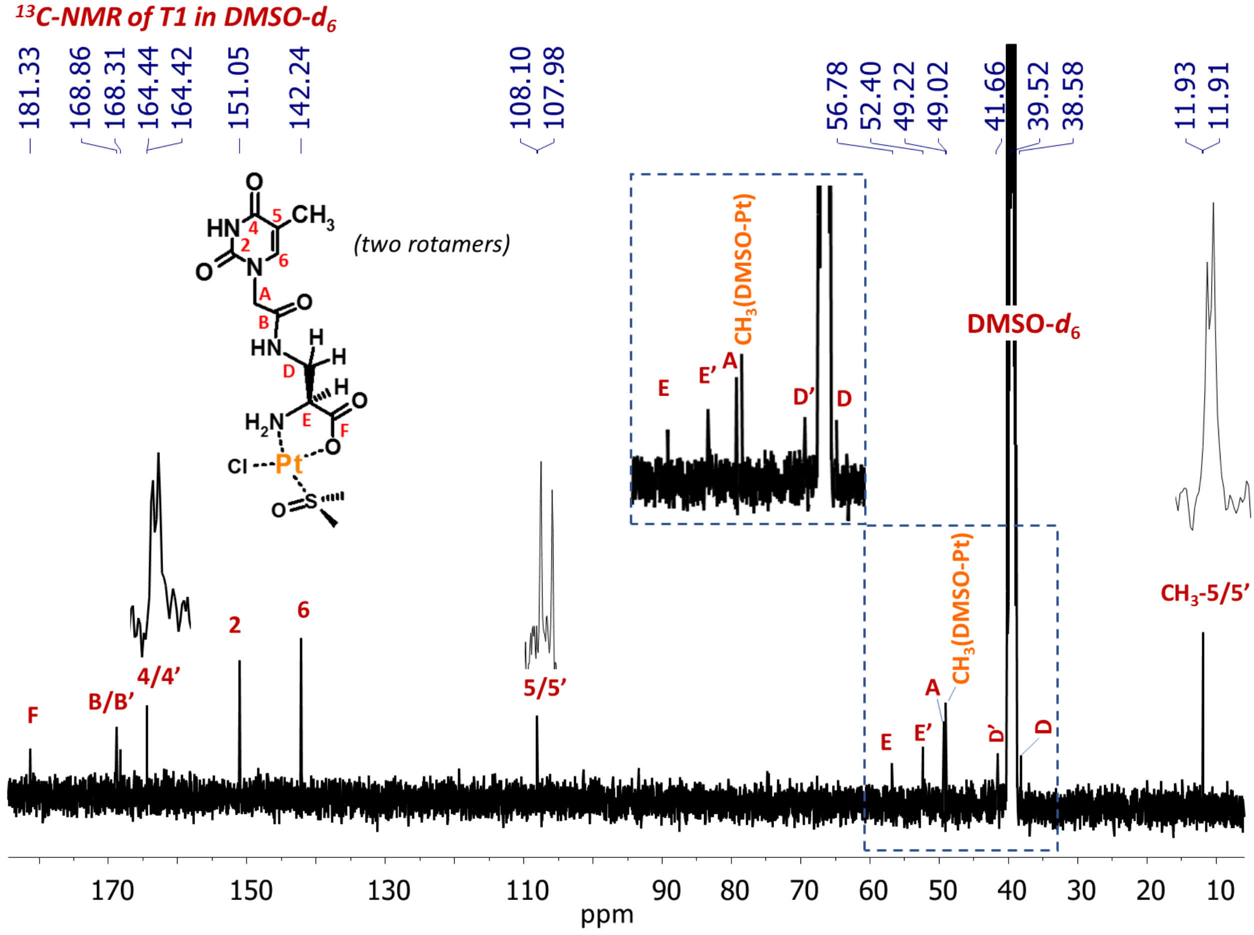

Figure S6: ^{1}H- (400 MHz) and ^{13}C-NMR (125 MHz) spectra of **T1** in DMSO-d$_6$. Enlargements of selected areas are also reported.

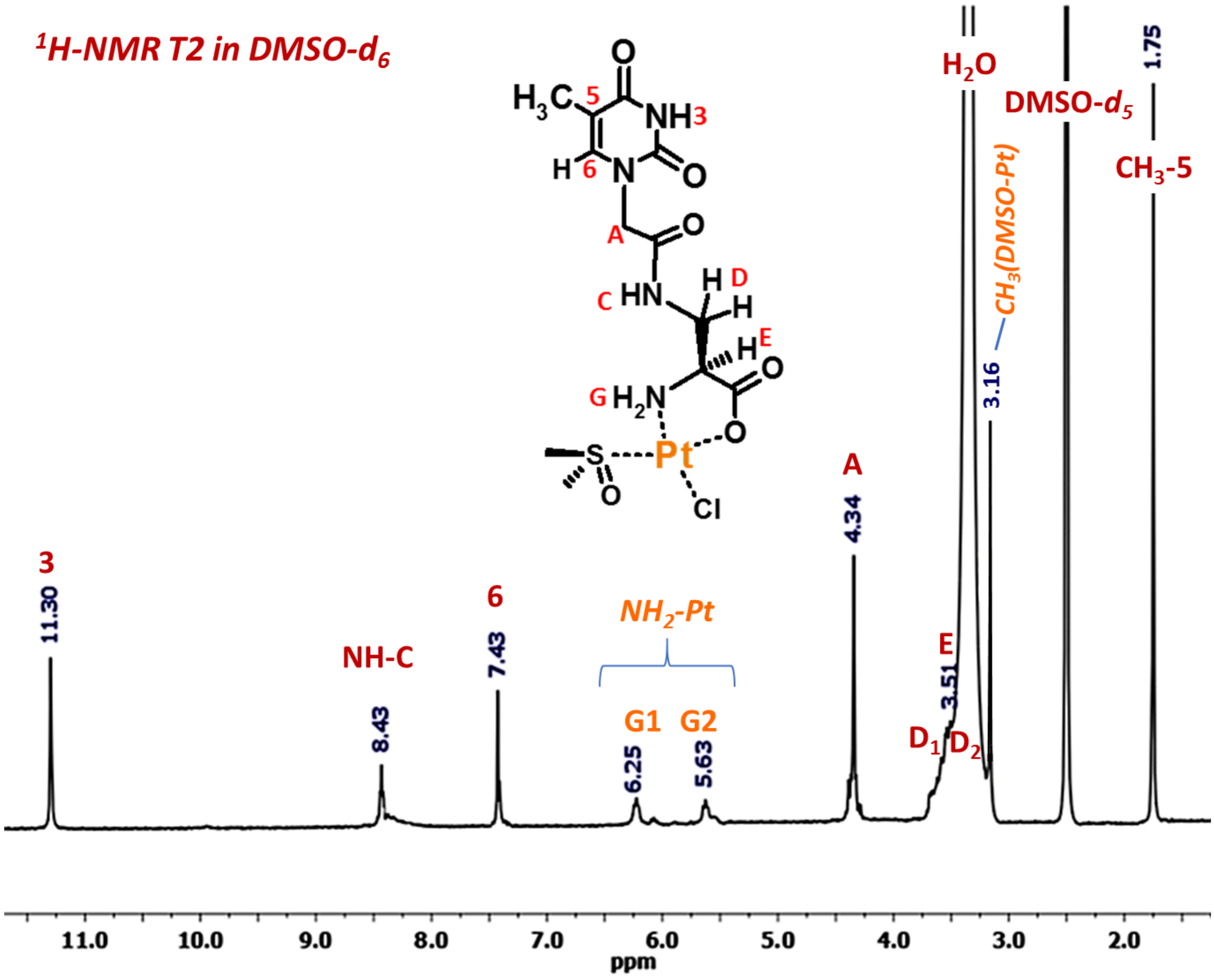

Figure S7:^{1}H-NMR spectrum (400 MHz) of **T2** in DMSO-d6.

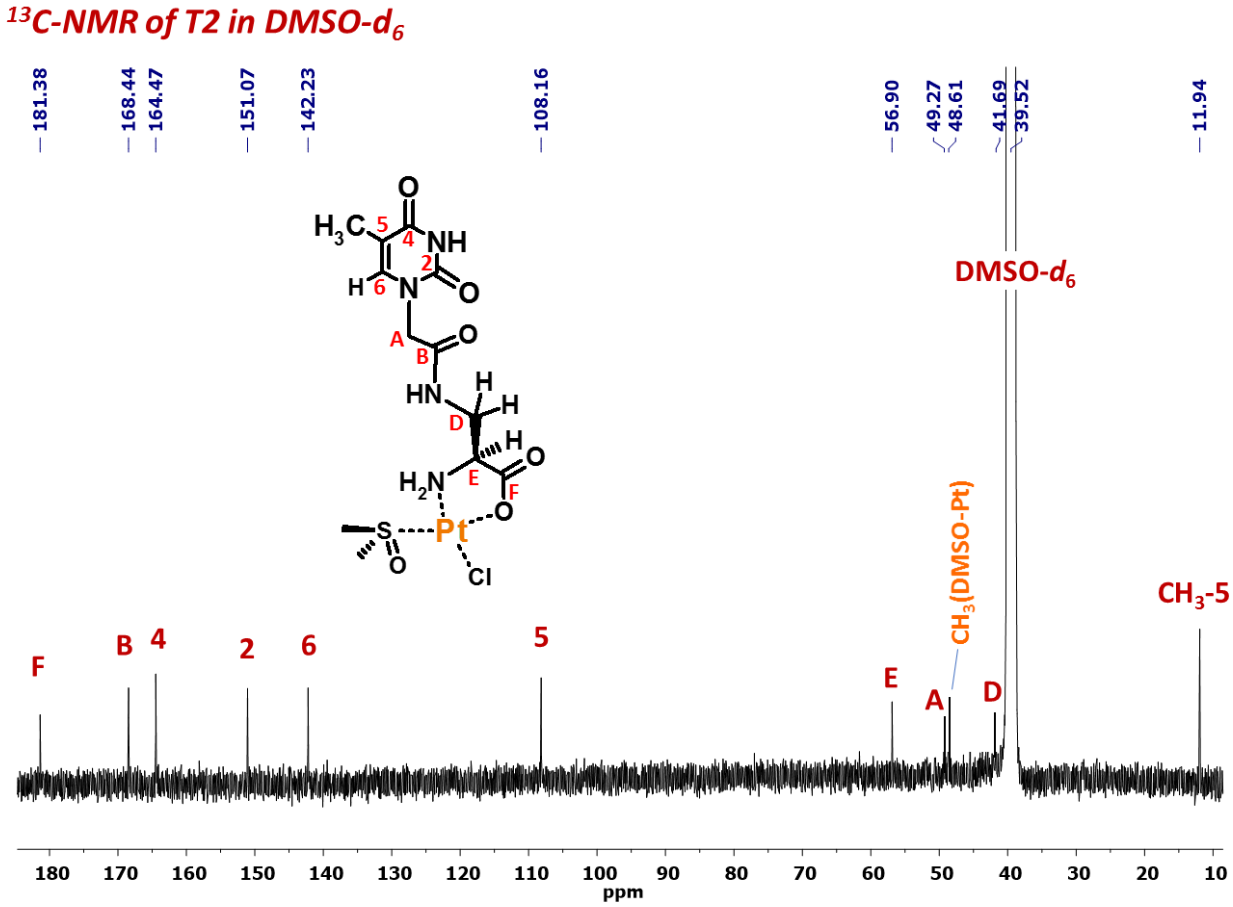

Figure S8: ^{13}C-NMR (100 MHz) spectrum of **T2** in DMSO-d_6.

Table S1: Direct ^{1}H → ^{13}C correlations based on the observed cross peaks in the bidimensional HSQC spectra of DAP(T)-OH, **T1** and **T2**.

HSQC DAP(T)-OH	
**7.42 (H-6)→**	142.31 (C-6)
**4.32 (A) →**	49.16 (A)
**3.55 (D1),<br>3.33 (D2) →**	39.62 (D)
**3.30 (E) →**	54.04 (E)
**1.74 (CH_3-5)**	11.87 (C-5)

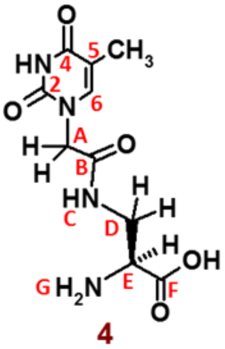

4

HSQC T1	
**7.44 (H-6)/<br>7.43 (H-6') →**	142.24 (C-6)
**4.35 (A) →**	49.22 (A)
**3.64 (D1),<br>3.49 (D2) →**	38.58 (D)
**3.56 (D1'),<br>3.28 (D2') →**	41.66(D')
**3.90 (E) →**	52.40 (E)
**3.52 (E') →**	56.78 (E')
**1.75 (CH_3-5) →**	11.93 (C-5)/<br>11.91 (C-5')
**3.16 (CH_3-DMSO)→**	49.02

HSQC T2	
**7.43 (H-6) →**	142.23 (C-6)
**4.34 (A) →**	49.27 (A)
**3.55 (D1),<br>3.29 (D2) →**	41.69 (D)
**3.51 (E) →**	56.90 (E)
**1.75 (CH_3-5) →**	11.94 (C-5)
**3.16 (CH_3-DMSO)→**	48.61

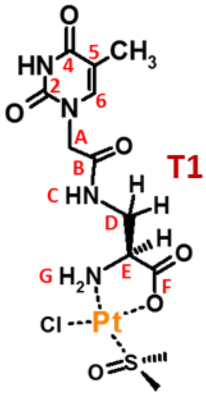

T1

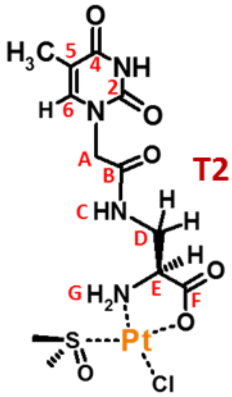

T2

Table S2: ^{1}H → ^{1}H correlations based on the observed cross peaks in the bidimensional COSY spectra of DAP(T)-OH, **T1** and **T2**.

COSY DAP(T)-OH				
**8.39 (C) →**	3.55 (D1)	3.33 (D2)		
**7.42 (H-6)→**	1.74 (CH_3-5)			
**3.55 (D1) →**	8.39 (C)	3.33 (D2)	3.30 (E)	
**3.30 (E) →**	3.55 (D1)	3.33 (D2)		
**3.33 (D2)→**	8.39 (C)	3.55 (D1)	3.30 (E)	
**1.74 (CH_3-5) →**	7.42 (H-6)			

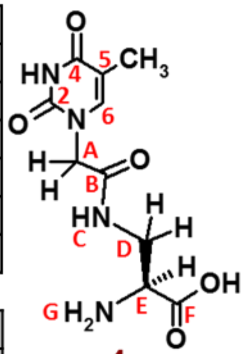

4

COSY T1				
**8.58 (C) →**	3.64 (D1)	3.49 (D2)		
**8.48 (C') →**	3.56 (D1')	3.28 (D2')		
**7.44 (H-6)/ 7.43 (H-6')→**	1.75 (CH_3-5)			
**6.29 (G1) →**	5.68 (G2)	3.90 (E)	3.52 (E')	
**5.68 (G2) →**	6.29 (G1)	3.90 (E)	3.52 (E')	
**3.90 (E) →**	6.29 (G1)	5.68 (G2)	3.64 (D1)	3.49 (D2)
**3.64 (D1) →**	8.58 (NH-C)	3.90 (E)	3.49 (D2)	
**3.56(D1') →**	8.48 (C')	3.52 (E')	3.28 (D2')	
**3.52 (E') →**	6.29 (G1)	5.68 (G2)	3.56 (D1')	3.28 (D2')
**3.49 (D2)→**	8.58 (C)	3.90 (E)	3.64 (D1)	
**3.28 (D2')→**	8.48 (C')	3.56 (D1')	3.52 (E')	
**1.75 (CH_3-5) →**	7.43/ 7.44 (H-6)			

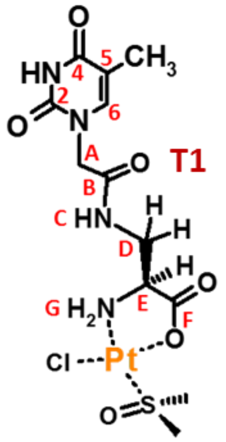

T1

COSY T2				
**8.43 (C) →**	3.55 (D1)	3.29 (D2)		
**7.43 (H-6)→**	1.75 (CH_3-5)			
**6.25 (G1) →**	5.63 (G2)	3.51 (E)		
**5.63 (G2) →**	6.25 (G1)	3.51 (E)		
**3.55 (D1) →**	8.43 (C)	3.51 (E)	3.29 (D2)	
**3.51 (E) →**	6.25 (G1)	5.63 (G2)	3.55 (D1)	3.29 (D2)
**3.29 (D2)→**	8.43 (C)	3.55 (D1)	3.51 (E)	
**1.75 (CH_3-5) →**	7.43 (H-6)			

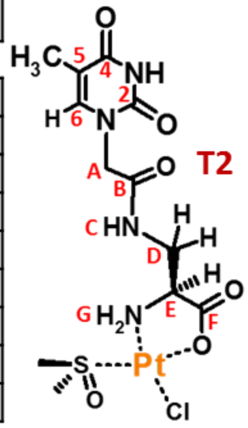

T2

Table S3: Long range $^{1}H \rightarrow ^{13}C$ correlations based on the observed cross peaks in the bidimensional HMBC spectra of **T1** and **T2**.

**HMBC T1**				
**11.29 (H-3)/ 11.28 (H-3')**	108.10 (C-5)/ 107.98 (C-5')			
**7.44 (H-6) 7.43 (H-6') →**	164.44 (C-4)/ 164.42 (C-4')	151.05 (C-2)	108.10 (C-5)/ 107.98 (C-5')	49.22 (A)
**4.35 (A) →**	168.85 (B)/ 168.31 (B')	151.05 (C-2)	142.24 (C-6)	
**3.49 (D2) →**	168.85 (B)	52.40 (E)		
**3.28 (D2') →**	168.31 (B')	56.78 (E')		
**1.75 (CH_3-5) →**	164.44 (C-4')/ 164.42 (C-4)	142.24 (C-6)	108.10 (C-5)/ 107.98 (C-5')	

**HMBC T2**				
**11.30 (H-3)**	108.16 (C-5)			
**7.43 (H-6) →**	164.47 (C-4)	151.07 (C-2)	108.16 (C-5)	49.27 (A)
**4.34 (A) →**	168.44 (B)	151.07 (C-2)	142.23 (C-6)	
**3.29 (D2) →**	168.44 (B)	56.90 (E)		
**1.75 (CH_3-5) →**	164.47 (C-4)	142.23 (C-6)	108.16 (C-5)	

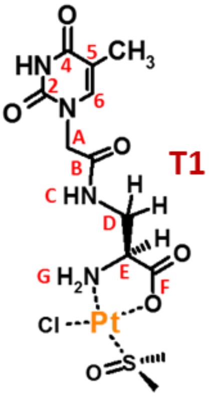

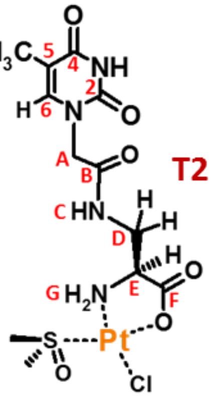

Table S4: $^1H \rightarrow {}^1H$ correlations based on the observed cross peaks in the bidimensional NOESY spectra of **T1** and **T2** (the red arrow indicates the correlation useful to discriminate the relative position of the DMSO and chloride ligands in **T2**).

NOESY T1				
**8.58 (C)/ 8.48 (C') →**	6.29 (G1), 5.68 (G2)	4.35 (A)		
**7.44 (H-6)/ 7.43 (H-6')→**	4.35 (A)	1.75 (CH_3-5)		
**6.29 (G1) →**	8.58 (C)/ 8.48 (C')	5.68 (G2)	3.52 (E')	3.90 (E)
**5.68 (G2) →**	8.58 (C)/ 8.48 (C')	6.29 (G1)	3.52 (E')	3.90 (E)
**4.35 (A)**	7.44 (H-6)/ 7.43 (H-6')			
**1.75 (CH_3-5) →**	7.44 (H-6)/ 7.43 (H-6')			

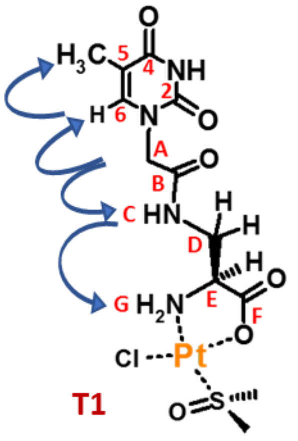

NOESY T2				
**8.43 (C) →**	6.25 (G1)	5.63 (G2)	4.34 (A)	3.55 (D1)
**7.43 (H-6)→**	4.34 (A)	1.75 (CH_3-5)		
**6.25 (G1) →**	8.43 (C)	5.63 (G2)	3.51 (E)	**3.16 (CH_3-DMSO)**
**5.63 (G2) →**	8.43 (C)	6.25 (G1)	3.51 (E)	
**4.33 (A) →**	8.43 (C)	7.44 (H-6)		
**1.75 (CH_3-5) →**	7.43 (H-6)			

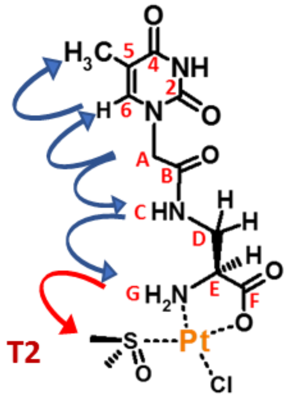

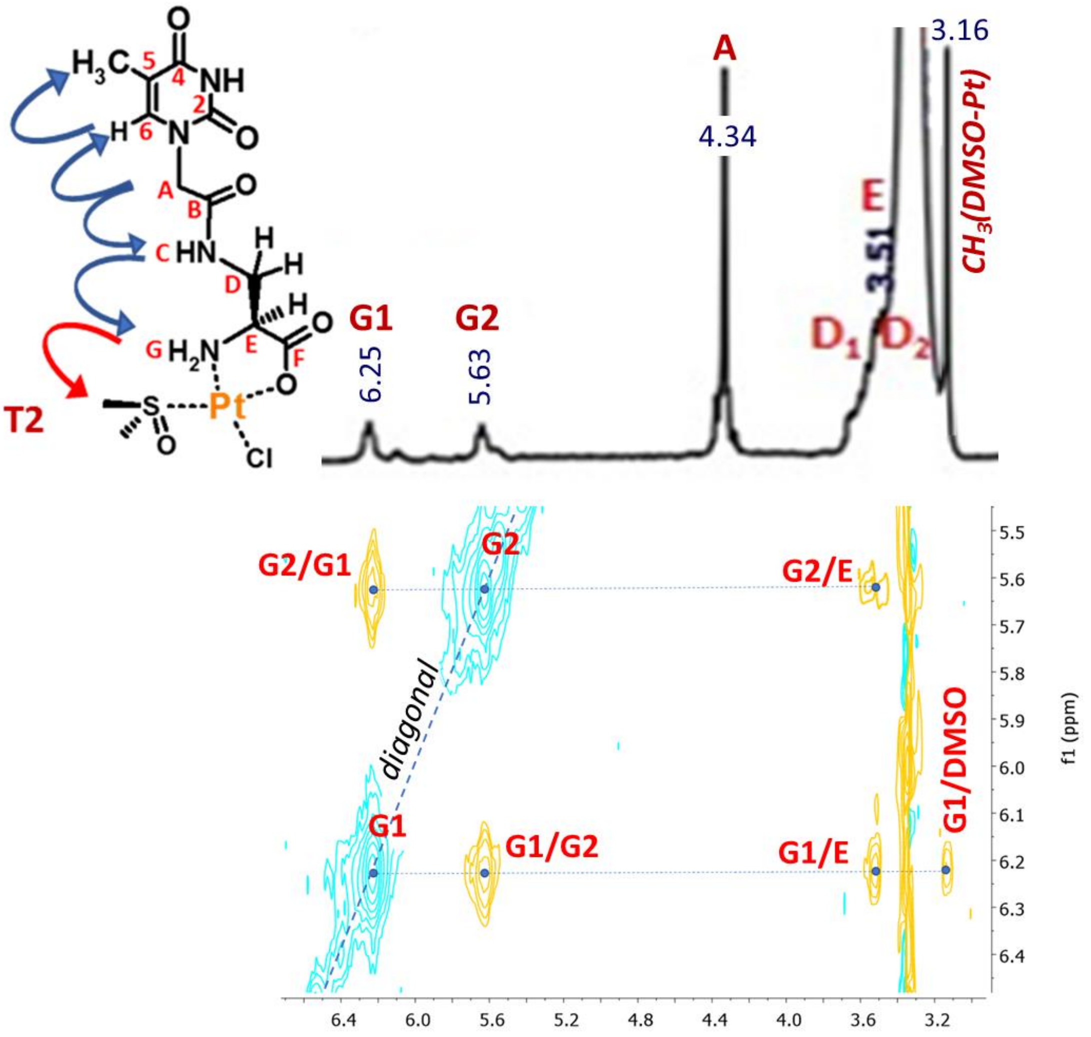

Figure S9: Enlargement of the NOESY spectrum (400 MHz) of **T2** in DMSO-d_6.

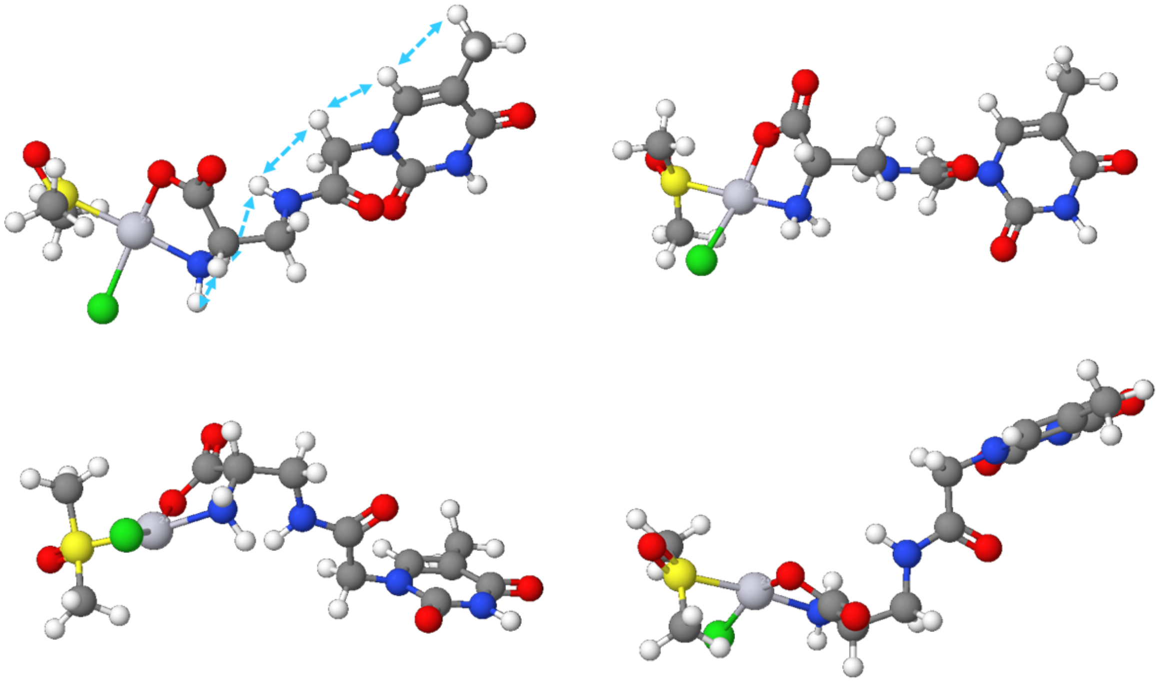

Figure S10: Energy-minimized three dimensional structure of **T1** {isomeric SMILES CC1C(=O)N([H])C(=O)N(CC(N([H])CC2C(=O)O[Pt+2](Cl)(S(=O)(C)C)N2([H])[H])=O)C=1}: 3D structure images (random low energy conformers) realized by MOLVIEW (http://molview.org). Light blue dashed arrows highlighted the spatial proximity of the corresponding protons, as also experimentally evidenced in the NOESY spectra (Table S4).

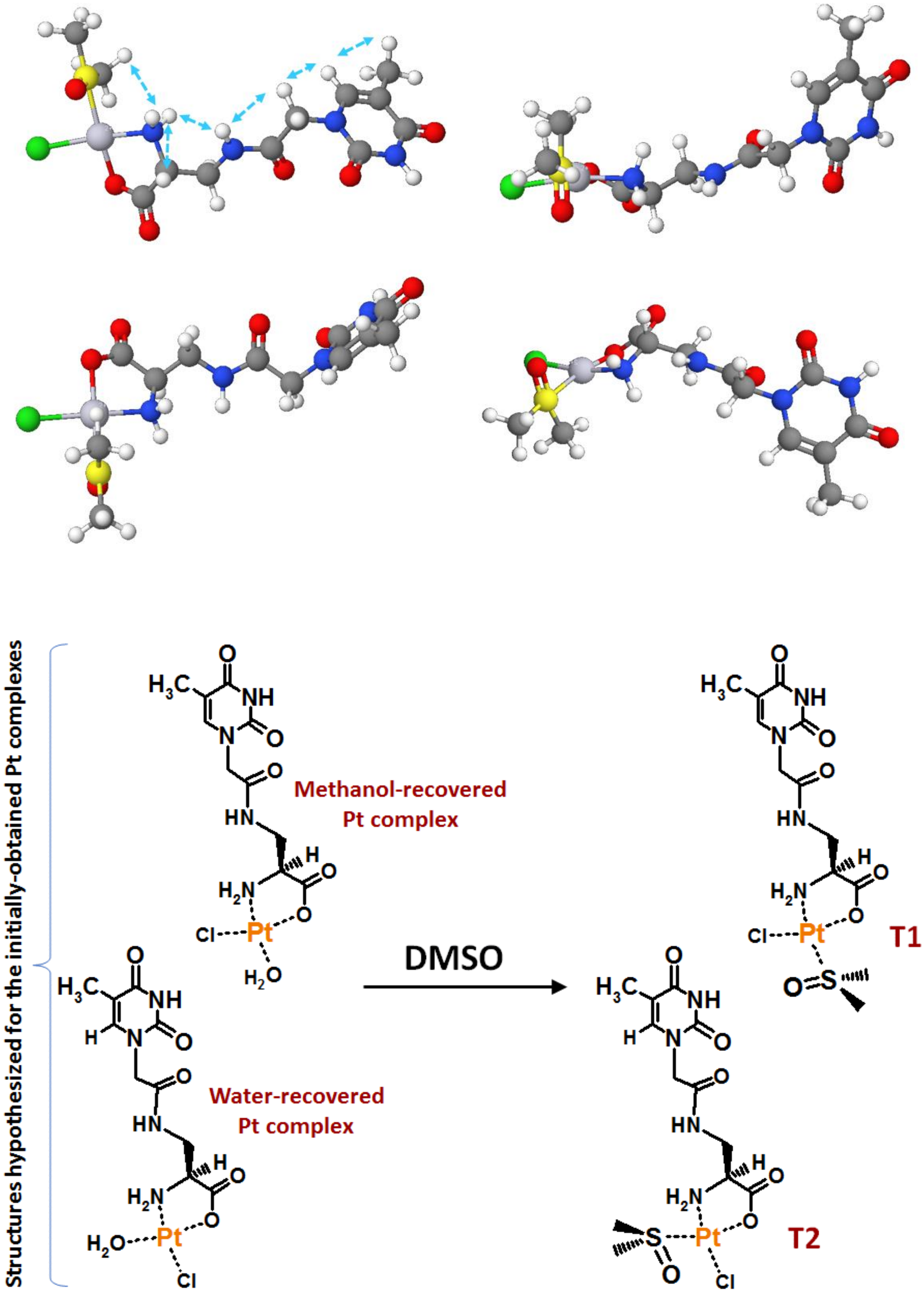

Figure S11: Energy-minimized three dimensional structure of **T2** (up) {isomeric SMILES CC1=CN(CC(=O)N(CC2N([H])([H])[Pt+2](Cl)(S(C)(C)=O)OC2=O)[H])C(=O)N([H])C1=O}: 3D structure images (random low energy conformers) realized by MOLVIEW (http://molview.org). Light blue dashed arrows highlighted the spatial proximity of the corresponding protons, as also experimentally evidenced in the NOESY spectra (see Table S4). Final structures of **T1** and **T2** and hypothesized ones for their initially-formed precursors, the methanol- and water-recovered Pt complexes (bottom).

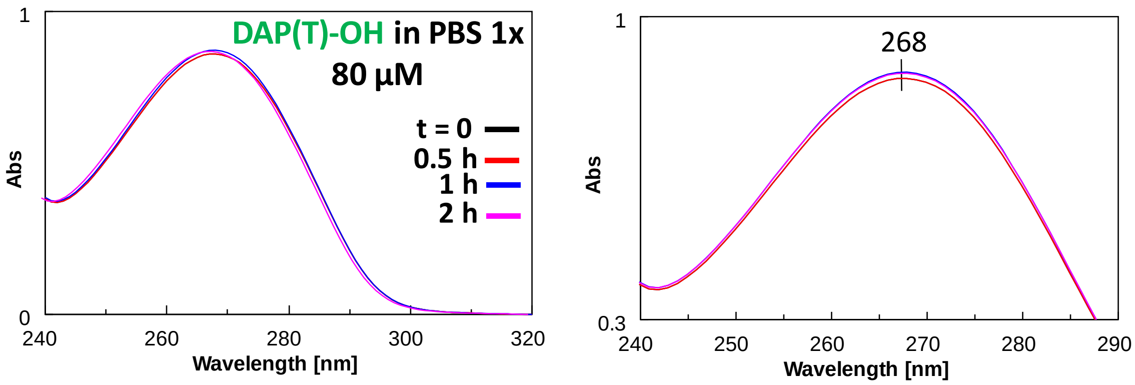

Figure S12: UV-vis spectra of DAP(T)-OH in PBS monitored over time (for clarity, only the overlapped spectra of the first 2 h monitoring were shown).

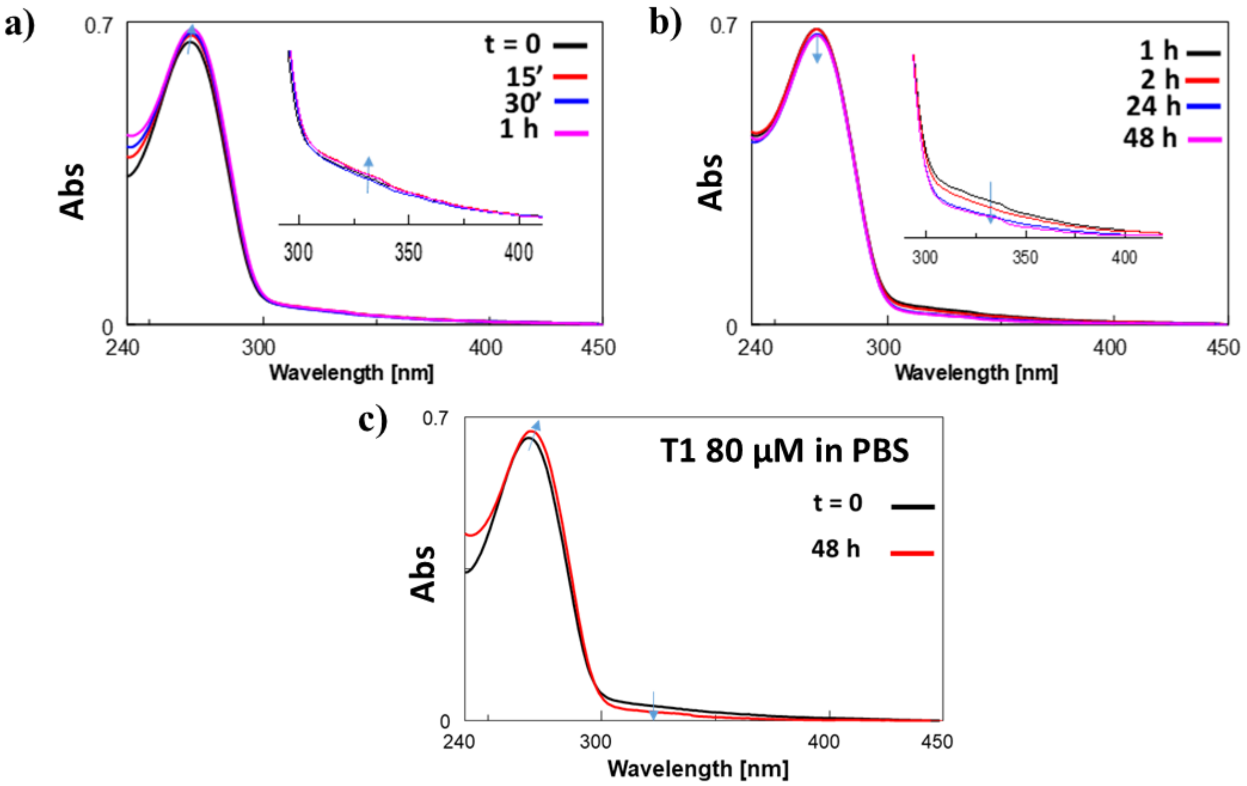

Figure S13: a,b) UV-vis spectra of **T1** in PBS at 80 µM concentration recorded over time, up to 48 h. **c**) Overlapped UV-vis spectra of **T1** immediately after its dissolution in PBS and after 48 h (black and red lines, respectively).

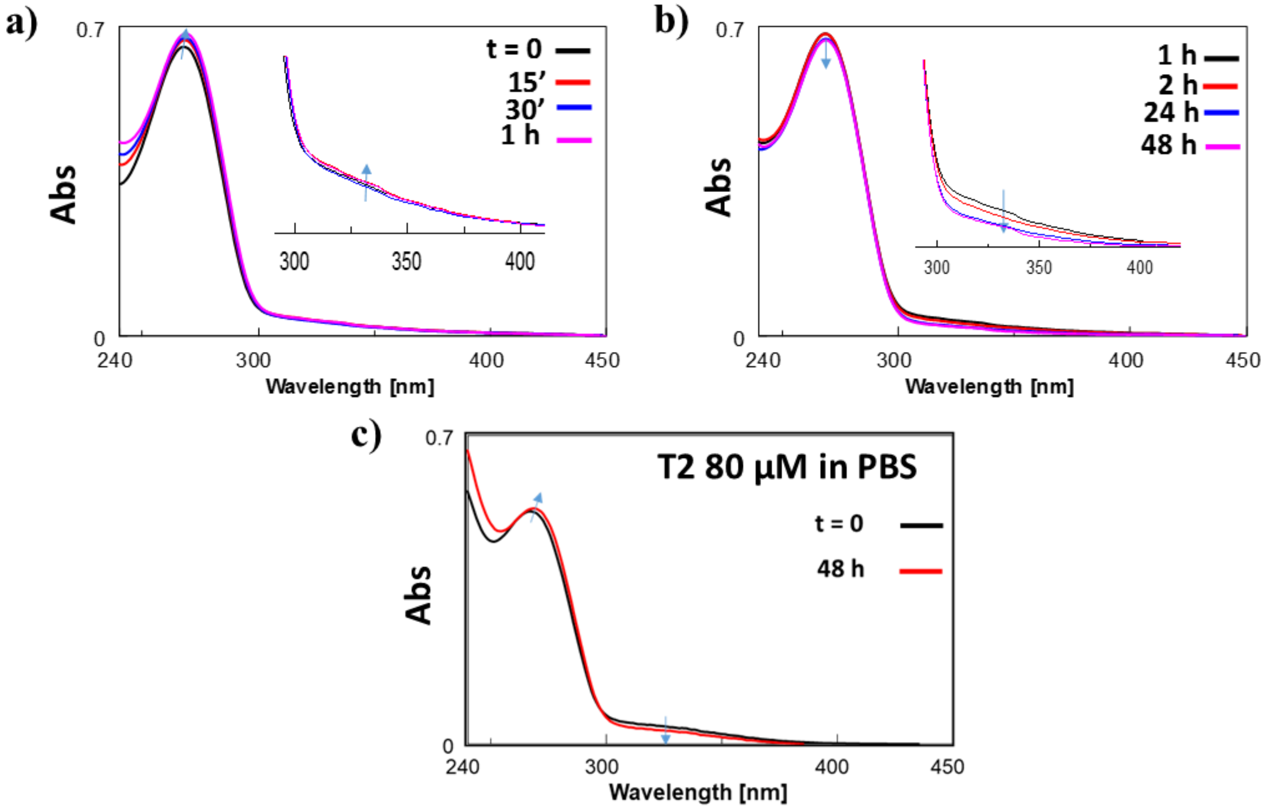

Figure S14: **a,b**) UV-vis spectra of **T2** in PBS at 80 µM concentration recorded over time, up to 48 h. **c**) Overlapped UV-vis spectra of **T2** immediately after its dissolution in PBS and after 48 h (black and red lines, respectively).

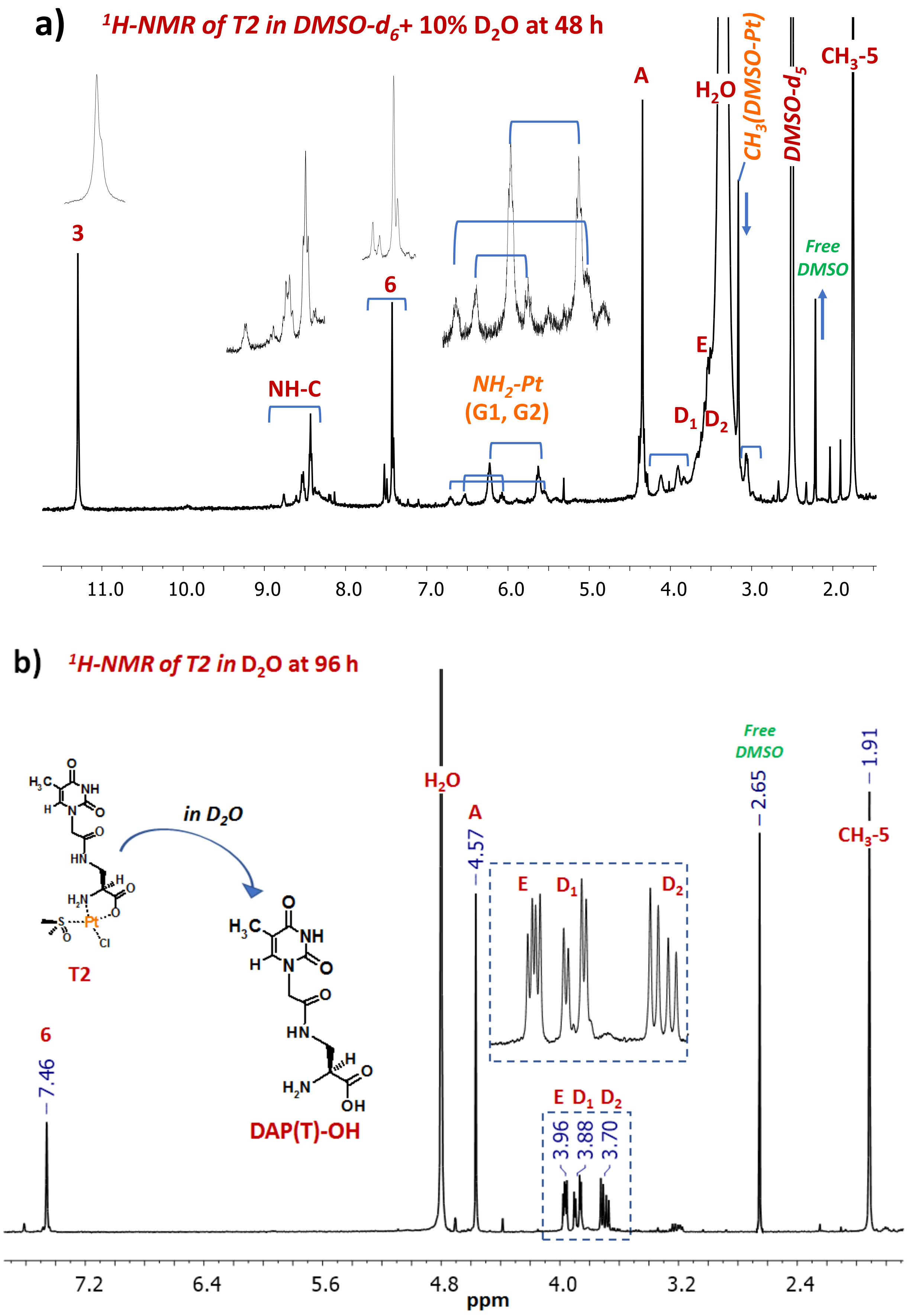

Figure S15: ^{1}H-NMR spectra (400 MHz) of **T2 a)** in DMSO-d$_6$, 48 h after the addition of 10% D_2O and **b)** 96 h after its dissolution in D_2O.

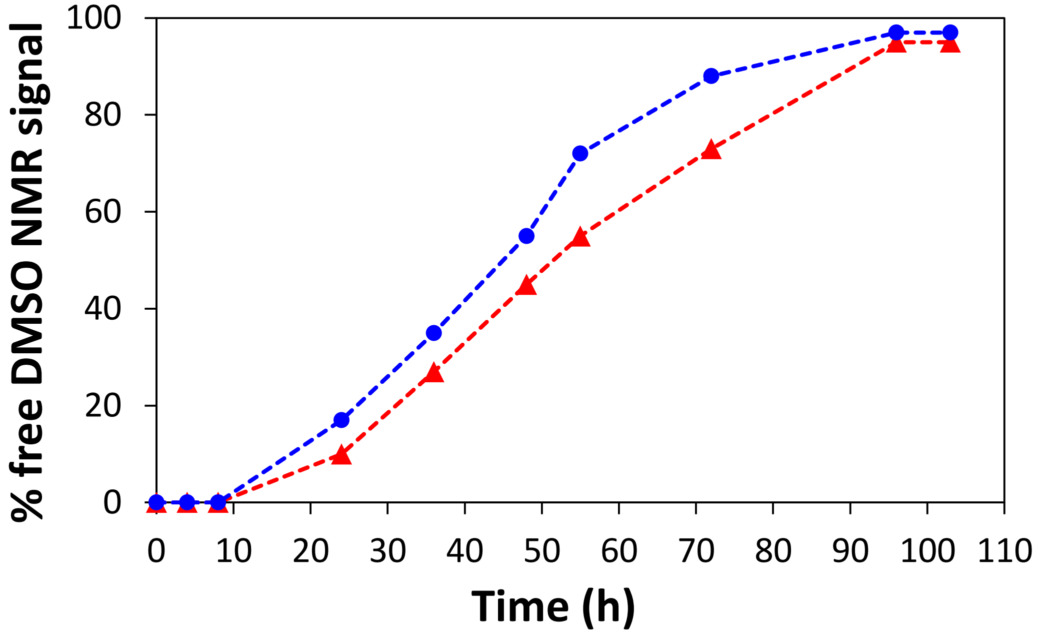

Figure S16: ^{1}H-NMR-time course experiments on **T1** and **T2** dissolved in D_2O. The % of the free DMSO NMR signal, compared to that of CH_3-5, was reported as a function of the time (h) after platinum complexes dissolution.

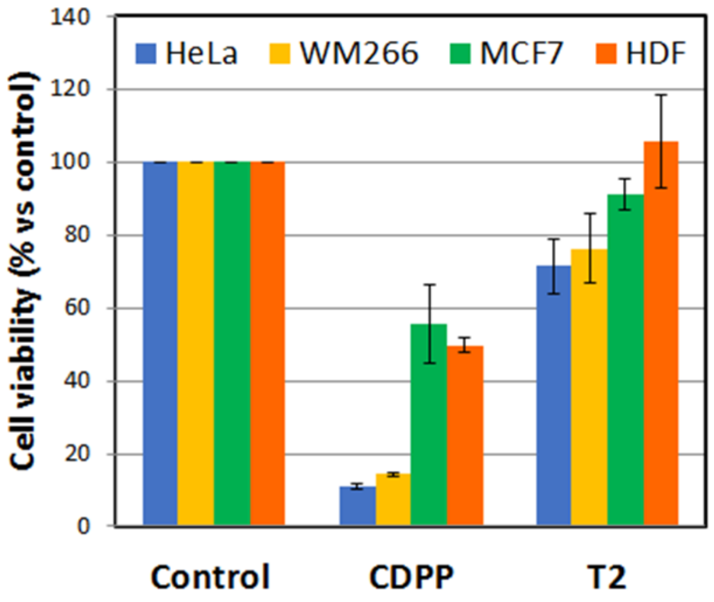

Figure S17: Comparison of HeLa, WM266, MCF-7 and HDF cell viability incubated with CDDP or **T2** at 25 µM concentration at 37 °C for 48 h. Cell viability was measured by using the MTT assay. The results are presented as the percentage of living cells with respect to the control (vehicle-treated cells) and are expressed as means ± SE of at least three independent experiments performed in triplicate.

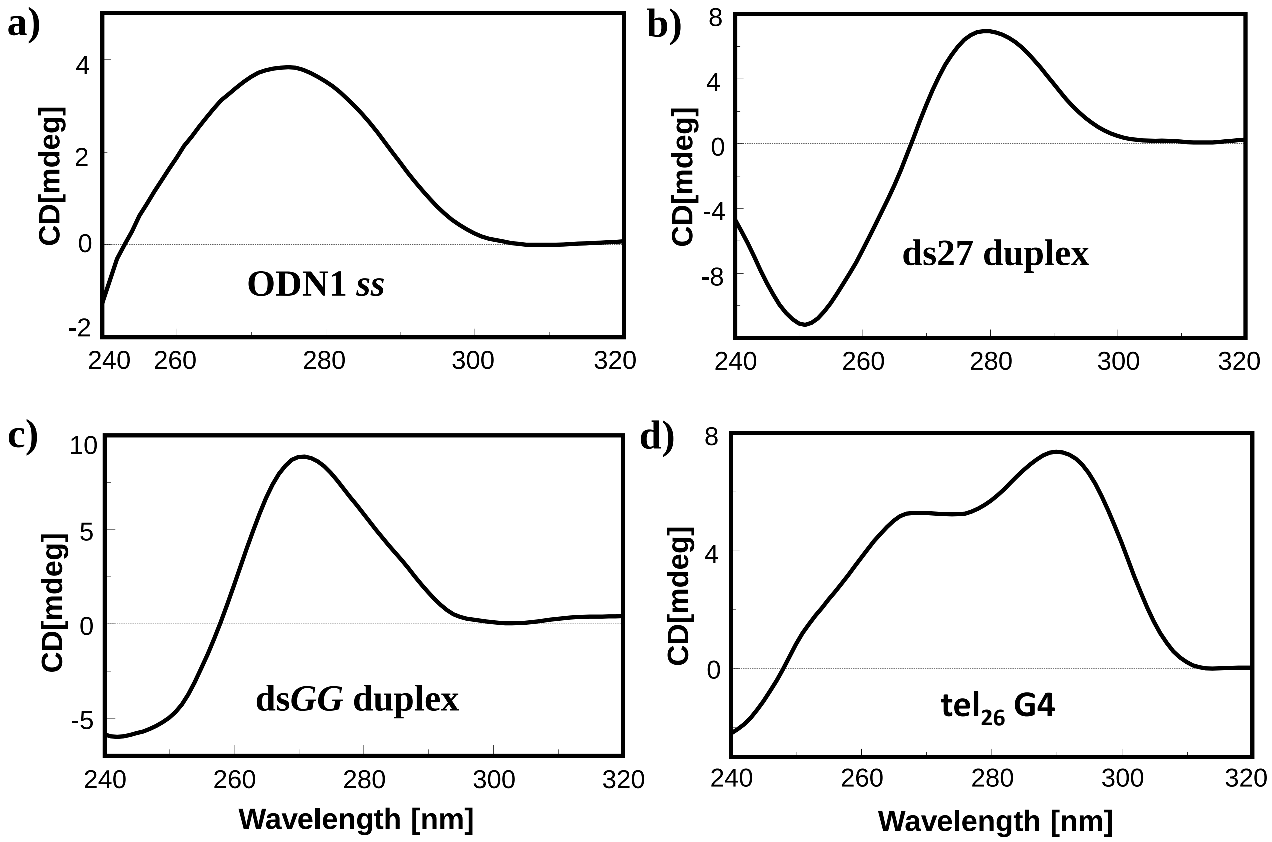

Figure S18: CD spectra of (**a**) the single strand ODN1, (**b**) the hairpin duplex ds27, (**c**) the hairpin duplex ds*GG*, and (**d**) the G-quadruplex tel_{26}. All the sequences were analysed at a 2 μM concentration in 100 mM KCl, 7 mM Na_2HPO_4/NaH_2PO_4, pH = 7.2, 20 °C (optical path = 1 cm).

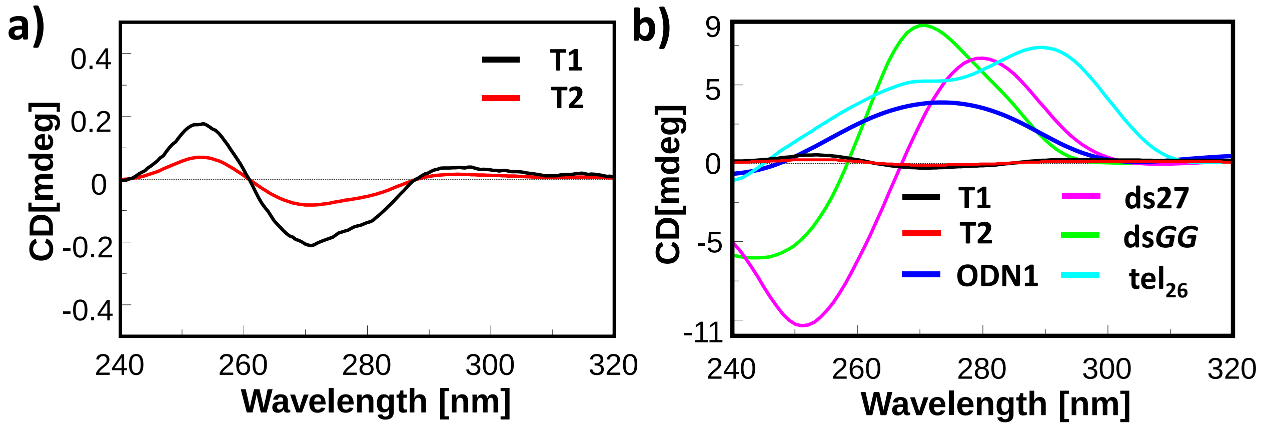

Figure S19: Overlapped CD spectra of **T1** and **T2** at 20 μM concentration in 100 mM KCl, 7 mM Na_2HPO_4/NaH_2PO_4, pH = 7.2, at 20 °C, (**a**) alone or (**b**) in comparison with the indicated DNA systems, each at 2 μM concentration (optical path = 1 cm).

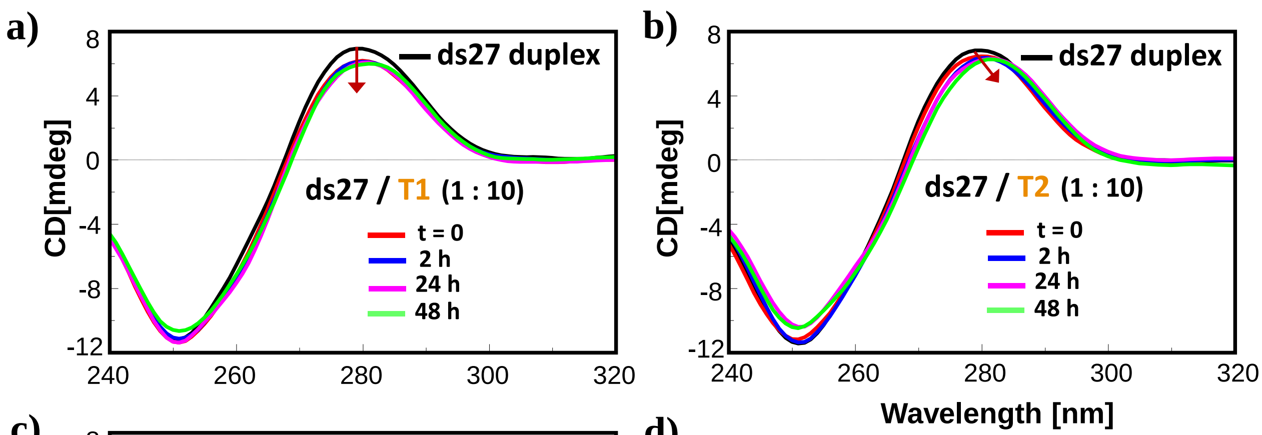

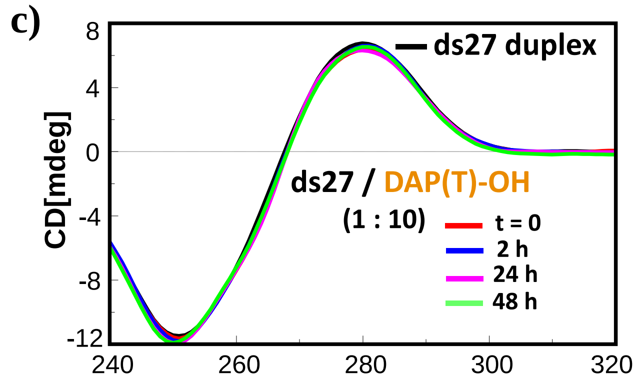

d)

System	ΔCD_{280}
ds27 / T1	1.030
ds27 / T2	0.325
ds27 / DAP(T)-OH	0.024

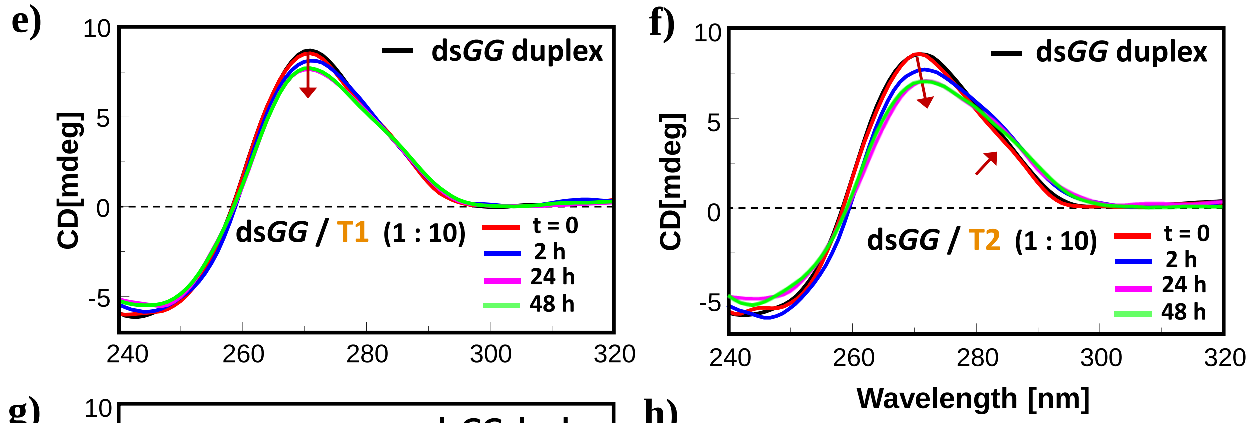

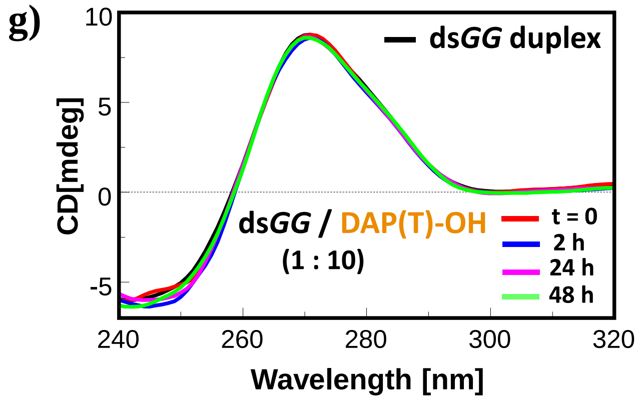

h)

System	ΔCD_{271}
ds*GG* / T1	0.995
ds*GG* / T2	1.393
ds*GG* / DAP(T)-OH	0.140

Figure S20: Overlapped CD spectra of the hairpin duplexes ds27 (**a-c**) and ds*GG* (**e-g**), each at 2 µM concentration, in the absence (black lines) and presence of **T1** (**a** and **e**), **T2** (**b** and **f**) and DAP(T)-OH (**c** and **g**) (20 µM each) at different times (0, 2, 24, 48 h) after the addition of the target molecules. Differences in the CD signal at 280 (for ds27) and 271 (for ds*GG*) nm between the CD spectra of the duplex before and after 48 h incubation with each added compound (**d** and **h**) (optical path = 1 cm).

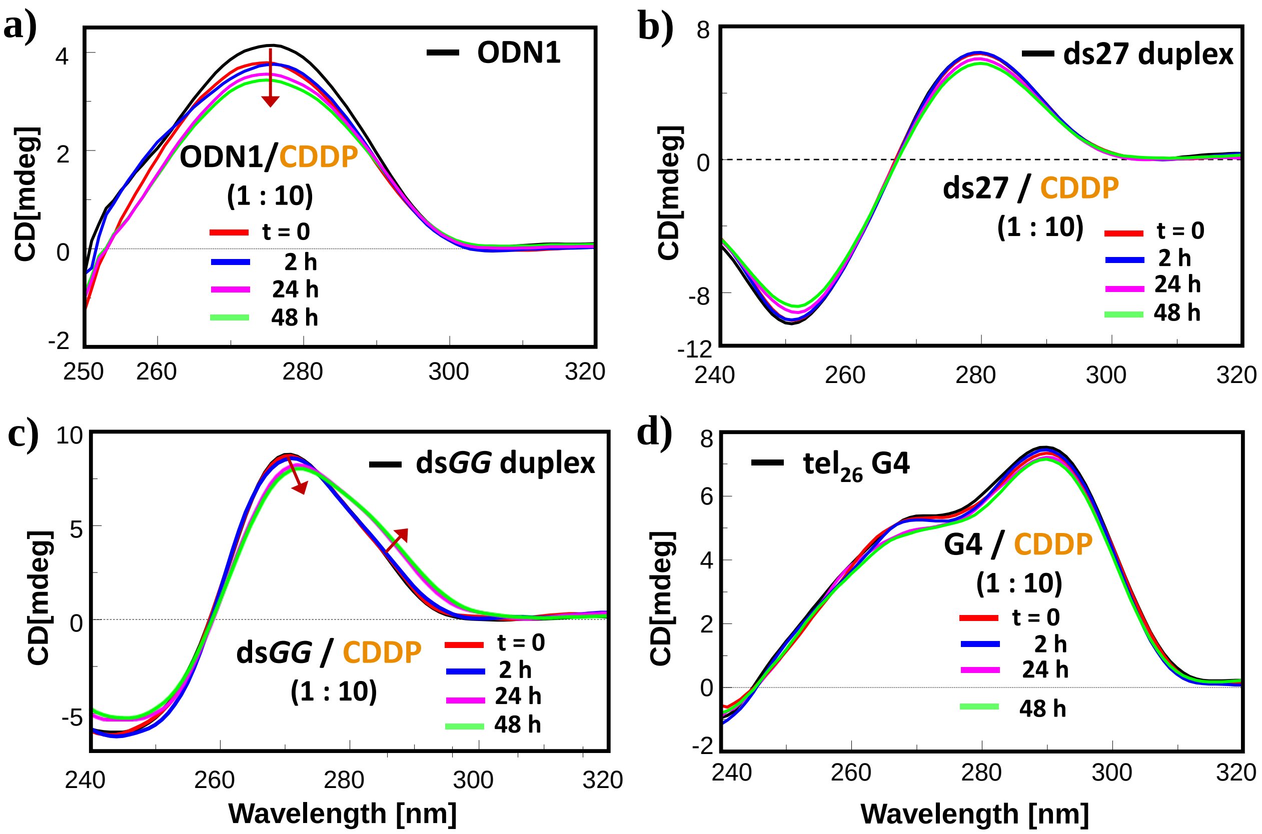

Figure S21: Overlapped CD spectra of the DNA systems (2 µM) in the absence (black lines) and presence of CDDP (20 µM) at different times (0, 2, 24, 48 h) after its addition: **a)** ODN1, **b)** hairpin duplex ds27, **c)** hairpin duplex ds*GG*, **d)** tel_{26}. The experiments were carried out in 100 mM KCl, 7 mM Na_2HPO_4/NaH_2PO_4, pH = 7.2, at 20 °C (optical path = 1 cm).

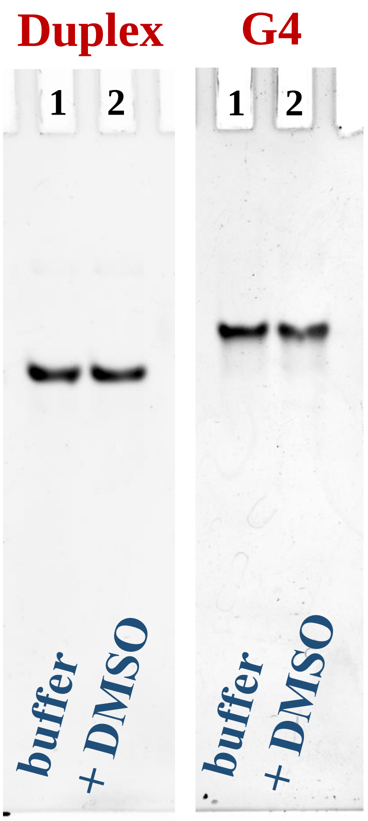

Figure S22: Representative 25 % polyacrylamide gel electrophoresis (PAGE) under native conditions relative to the *GG*-duplex (ds*GG*) and G-quadruplex DNA systems (2 µM concentration), in only buffer (100 mM KCl, 7 mM Na_2HPO_4/NaH_2PO_4, pH = 7.2) (lanes 1), or incubated for 48 h with 5 % DMSO (lanes 2). Gel was run at 80 V, at r.t. for 3.3 h in TBE 1X buffer.

Abbreviations: CD (circular dichroism), COSY (correlation spectroscopy), DAP (2,3-diaminopropanoic acid), DMSO (dimethyl sulfoxide), ESI (electrospray ionization), G4 (G-quadruplex), HMBC (heteronuclear multiple-bond correlation), HSQC (heteronuclear single quantum correlation), LC (liquid chromatography), MALDI (matrix-assisted laser desorption ionization), MS (mass spectrometry), NMR (nuclear magnetic resonance), NOESY (nuclear Overhauser effect spectroscopy), ODN (oligodeoxyribonucleotide), PBS (phosphate-buffered saline), ppm (parts per million), TFA (trifluoroacetic acid), TIC (total ion current), TOF (time of flight), t_R (retention time).
